# Yu-Ping-Feng Formula Ameliorates Alveolar-Capillary Barrier Injury Induced by Exhausted-Exercise *via* Regulation of Cytoskeleton

**DOI:** 10.3389/fphar.2022.891802

**Published:** 2022-06-24

**Authors:** Di Wang, Quan Li, Chun-Shui Pan, Li Yan, Kai Sun, Xiao-Yi Wang, Gulinigaer Anwaier, Qian-Zan Liao, Ting-Ting Xie, Jing-Yu Fan, Xin-Mei Huo, Yuan Wang, Jing-Yan Han

**Affiliations:** ^1^ Department of Integration of Chinese and Western Medicine, School of Basic Medical Sciences, Peking University Health Science Center, Beijing, China; ^2^ Tasly Microcirculation Research Center, Peking University Health Science Center, Beijing, China; ^3^ Key Laboratory of Microcirculation, State Administration of Traditional Chinese Medicine of the People’s Republic of China, Beijing, China; ^4^ Key Laboratory of Stasis and Phlegm, State Administration of Traditional Chinese Medicine of the People’s Republic of China, Beijing, China; ^5^ State Key Laboratory of Core Technology in Innovative Chinese Medicine, Tianjin, China; ^6^ State Key Laboratory of Natural and Biomimetic Drugs, School of Pharmaceutical Sciences, Peking University, Beijing, China

**Keywords:** traditional Chinese medicine, lung injury, proteomics, cell junctions, stress fiber

## Abstract

**Background:** Yu-ping-feng powder (YPF) is a compound traditional Chinese medicine extensively used in China for respiratory diseases. However, the role of YPF in alveolar-capillary barrier dysfunction remains unknown. This study aimed to explore the effect and potential mechanism of YPF on alveolar-capillary barrier injury induced by exhausted exercise.

**Methods:** Male Sprague–Dawley rats were used to establish an exhausted-exercise model by using a motorized rodent treadmill. YPF at doses of 2.18 g/kg was administrated by gavage before exercise training for 10 consecutive days. Food intake-weight/body weight, blood gas analysis, lung water percent content, BALF protein concentration, morphological observation, quantitative proteomics, real-time PCR, and Western blot were performed. A rat pulmonary microvascular endothelial cell line (PMVEC) subjected to hypoxia was applied for assessing the related mechanism.

**Results:** YPF attenuated the decrease of food intake weight/body weight, improved lung swelling and hemorrhage, alleviated the increase of lung water percent content and BALF protein concentration, and inhibited the impairment of lung morphology. In addition, YPF increased the expression of claudin 3, claudin 18, occludin, VE-cadherin, and β-catenin, attenuated the epithelial and endothelial hyperpermeability *in vivo* and/or *in vitro*, and the stress fiber formation in PMVECs after hypoxia. Quantitative proteomics discovered that the effect of YPF implicated the Siah2-ubiquitin-proteasomal pathway, Gng12-PAK1-MLCK, and RhoA/ROCK, which was further confirmed by Western blot. Data are available via ProteomeXchange with identifier PXD032737.

**Conclusion:** YPF ameliorated alveolar-capillary barrier injury induced by exhausted exercise, which is accounted for at least partly by the regulation of cytoskeleton.

## Introduction

Although regular exercise benefits physical and mental health (Flynn and McFarlin, 2006; Gleeson et al., 2011; Rigonato-Oliveira et al., 2018), exhausted exercise has proven deleterious, leading to a panel of consequences, such as cardiac hypertrophy (Huang et al., 2019), and prone to develop upper respiratory tract infections (Fahlman and Engels, 2005; Walsh et al., 2011) and pulmonary interstitial edema or lung edema (Caillaud et al., 1995; McKenzie et al., 2005; Zavorsky, 2007). However, the study on the mechanism of pulmonary edema induced by exhausted exercise is limited so far, not to mention the protective measure.

Yu-Ping-Feng-San (YPF) is a compound traditional Chinese medicine extensively prescribed to treat respiratory diseases such as upper respiratory tract infection (Song et al., 2016), asthma (Wang et al., 2020), allergic rhinitis (Liao et al., 2020), and chronic obstructive pulmonary disease (Ma et al., 2018), and contains *Astragalus mongholicus* Bunge, *Atractylodes macrocephala* Koidz., and *Saposhnikovia divaricata* (Turcz. ex Ledeb.) Schischk. Researchers have shown that YPF has effects of bidirectional immunomodulating (Song et al., 2013; Sun et al., 2017; Yao et al., 2020), anti-oxidation (Huang et al., 2020), and inhibiting tumor growth (Wang et al., 2019). However, the role of YPF in alveolar-capillary-barrier injury is unclear. The present research aimed to test the effect of YPF on alveolar-capillary barrier injury induced by exhausted exercise and the underlying mechanism.

## Materials and Methods

### Reagents

YPF granules were obtained from Guangzhou Yi Fang Pharmaceutical Co. Ltd. (Guangzhou, China), which consist of *Astragalus mongholicus* Bunge (huangqi) (40%), *Atractylodes macrocephala* Koidz. (baizhu) (40%), and *Saposhnikovia divaricata* (Turcz. ex Ledeb.) Schischk. (fangfeng) (20%). YPF was dissolved in ultrapure water for administration to animals. Antibodies against MLC (myosin light chain), phosphor-MLC (Ser 19), RhoA, Rock (rho-associated kinase), Mypt1 (myosin phosphatase target subunit 1), phosho-Mypt1 (Thr853), PAK1 (p21-activated kinase 1), phospho-PAK1 (Ser 199), VE-cadherin, β-catenin, and Pan-keratin were purchased from Cell Signaling Technology (Danvers, MA, United States). Antibodies against β-actin, claudin 3, claudin 5, claudin 18, occludin, CD31, E-cadherin, and MLCK (Myosin light chain kinase) were purchased from Abcam (Cambridge, UK). Antibodies against Siah2 (Siah E3 ubiquitin protein ligase 2) and Gng12 (G protein subunit gamma 12) were purchased from Thermo (Rockford, IL, United States). Antibodies against phospho-MLCK (Tyr471) were purchased from Immunoway (Plano, TX, United States). Phalloidin used to label F-actin was also purchased from Thermo (Rockford, IL, United States).

### Animal Models

Male Sprague–Dawley rats weighing 180–200 g were purchased from Beijing Charles River Laboratory Animal Technology Company Limited (Certificate No. SCXK (Jing) 2016-0006) and housed in an environment of a temperature of 23 ± 2°C and relative humidity of 40 ± 5% with a 12 h light/dark cycle. Animals were kept in individual cages and given standard laboratory diet and water. All experimental procedures were approved by the Peking University Biomedical Ethics Committee Experimental Animal Ethics Branch (LA2018232), complying with the “Guidelines for the Care and Use of Laboratory Animals,” published by the National Institutes of Health.

The animal model was established as previously reported with a slight modification (Huang et al., 2019). Rats were first trained on a motorized rodent treadmill (YLS-15A; Dongguan BOZHI FAR Biotechnology Development, Guangdong, China) for 2 days, 60 min/day, 15 m/min. Rats showing adaptation to treadmill training were selected and randomized into four groups: the sham group, sham + YPF (2.18 g/kg) group, exhausted-exercise group, and exhausted-exercise + YPF (2.18 g/kg) group. Animals in the exhausted-exercise group and the exhausted-exercise + YPF group underwent treadmill training for 10 days, 60 min/day, keeping the treadmill speed set successively at 20, 20, 20, 25, 25, 25, 25, 30, 30, and 30 m/min. During the exhausted-exercise protocol, mild electric stimuli (1 mA, 3 Hz) were given from the back of the treadmill chamber to promote learning of running behavior. Animals in the sham group and the sham + YPF group were exempted from treadmill training after treadmill adaptation. Rats in the sham + YPF group and the exhausted-exercise + YPF group were administrated with YPF for 10 days by gavage at the dose of 2.18 g/kg. Animals in the sham group and the exhausted-exercise group were given an equal amount of ultrapure water (10 ml/kg) the same way for 10 days. The arterial blood gas analysis and euthanasia were conducted 2 h post the last dose of YPF administration.

### Preparation of Yu-Ping-Feng-Containing Serum

Male Sprague-Dawley rats were divided into control group and YPF group, and were administered by gavage with ultrapure water (10 ml/kg) or YPF (2.18 g/kg) twice a day for 3 days respectively. One hour after the final dose, blood samples were collected from the abdominal aorta when the rats were under anesthesia with 2% pentobarbital (60 mg/kg). The blood samples were centrifuged at 1,000 g for 15 min at 4°C, the serum was collected, sterile-filtered through a 0.22 μm microporous membrane filter, and stored at −80°C before use (Wang et al., 2019).

### Cell Culture

PMVEC, the pulmonary microvascular endothelial cell line (American Type Culture Collection, Rockville, MD, United States), was used for the study of underlying mechanisms (Wojciak-Stothard et al., 2006; Zhong et al., 2021). Cells were cultured in DMEM (Invitrogen, Grand Island, NY, United States) containing 10% fetal bovine serum at 37°C and humidified with 95% air-5% CO_2_. The cells were used between passages 7 and 9 for all experiments. PMVECs were seeded in 6-well cell culture plates and underwent serum starvation for 12 h once growing to 70% confluence. The cells were then divided into three groups: control + NORM serum, hypoxia + NORM serum, and hypoxia + YPF serum group. The cells in the control + NORM serum and the hypoxia + NORM serum were incubated with DMEM containing 12.5% control rat serum, while cells in the hypoxia + YPF serum group were incubated with DMEM containing 12.5% YPF rat serum for 1 h in normoxic conditions (20% O_2_-5% CO_2_-75% N_2_). Then cells in the hypoxia + NORM serum and the hypoxia + YPF serum group were exposed to hypoxia (1% O_2_-5% CO_2_-94% N_2_) for 4 h.

### Cell Viability Assay

PMVECs were seeded into a 96-well at a density of 5 × 10^4^ cells/well, incubated for 24 h with DMEM containing 10% FBS at 37°C in a 5% CO_2_ incubator, and then the cells underwent serum starvation for 12 h. Following washing with phosphate-buffered saline solution (PBS) twice, the cells were incubated with YPF-containing serum (6.25–100%) for 24 h. Then PMVEC cell viability was determined colorimetrically using Cell Counting Kit-8 assay as per the manufacturer’s instructions (DOJINDO, Kumamoto, Japan).

### Food-Intake Weight

Food-intake weight (FIW) of an animal was determined by the food weight of the day subtracting the food weight of the previous day in the cage.

### Blood Gas Analysis

After the exhausted-exercise protocol, the rats were sacrificed and blood was taken from the abdominal aorta. A blood-gas analyzer (ABL80 FLEX, Radiometer, California, United States) was used for detecting PaO_2_, SaO_2_, and PaCO_2_.

### Lung Water Percent Content and Protein Content in Bronchial Alveolar Lavage Fluid

The lung water percent content and protein level in the bronchial alveolar lavage fluid (BALF) were determined after finishing the exhausted-exercise protocol. Following harvesting the middle lobe of the right lung for histology, the remaining right lung was quickly weighed followed by drying at 60°C for 96 h to a constant weight. The lung water percent content was calculated by the formula: lung water percent content = (wet weight-dry weight)/wet weight × 100% (Zhou et al., 2017). For the determination of the protein content of BALF, the rats were inserted with a plastic cannula into the trachea and douched with 4 ml pre-cold sterile saline three times. BALF samples were centrifuged for 15 min at 4°C and the supernatants were collected (Zhou et al., 2017). The protein level in BALF was quantitated by a bicinchoninic acid assay method (Applygen, Beijing, China).

### Histology and Immunofluorescent Staining

At the end of the experiment, the middle lobe of the right lung was harvested from the rats, fixed in 4% paraformaldehyde for 48 h, and processed for paraffin sections (5 μm) as routine. Sections were stained with hematoxylin and eosin (H&E) to evaluate histology changes in the lungs with a light microscope (BX512DP70, Olympus, Tokyo, Japan). The thickness of the alveolar septum and alveolar area were measured in randomly selected five fields using Image-Pro Plus 6.0 software (Media Cybernetics, Bethesda, MD, United States) (Szoták-Ajtay et al., 2020), taking the average as the result. The perivascular edema index was calculated by the perivascular edema area divided by the vascular area. (Samano et al., 2009) using Image-Pro Plus 6.0 software.

Immunofluorescence staining was conducted on the sections as routine. Specifically, after antigen retrieval and blocking of nonspecific binding, the following primary antibodies were applied to the sections: mouse anti-claudin 5 (1:50), rabbit anti-claudin 3 (1:100), rabbit anti-claudin 18 (1:100), mouse anti-Pan-keratin (1:200), mouse anti-E-cadherin (1:100), and rabbit anti-CD31 (1:200) and incubated at 4°C overnight. After washing with PBS three times, the specific binding was recognized by the following secondary antibodies: Dylight 594-labeled goat anti-mouse IgG (1:100, KPL, Gaithersburg, MD, United States), Dylight 488-labeled goat anti-mouse IgG (1:100, KPL, Gaithersburg, MD, United States), and Cy3-labeled goat anti-rabbit IgG (1:100, KPL, Gaithersburg, MD, United States). The nuclei were displayed by Hoechst 3342 (molecular probe). The sections were observed by using laser scanning confocal microscope (TCS-SP8 STED 3X, Leica, Mannheim, Germany).

### F-actin Staining of the Cells

PMVECs fixed by 4% paraformaldehyde-fixed were permeabilized with 0.3% TritonX-100, stained with rhodamine-phalloidin for F-actin at 37°C for 2 h. Hoechst 3342 (molecular probes) was applied to stain all the nuclei. The results were examined by using a laser scanning confocal microscope (TCS-SP8 STED 3X, Leica, Mannheim, Germany).

### Intratracheal Instillation of the Polystyrene Microsphere

Intratracheal instillation of the polystyrene microsphere (1 mg/ml) was performed for rats from different groups under anesthesia with isoflurane in 70%N_2_/30%O_2_. After 6 h, the BALF was collected for detecting the percent of polystyrene microsphere per unit volume of BALF by flow cytometry (Gallios, Beckman, United States), and the middle lobe of the right lung and the liver were harvested for immunofluorescent staining. The polystyrene microsphere (PS, 2.5 ± 0.2 μm in diameter) was purchased from Ruixi Biological Technology Co. LTD. (Xian, China), which brings in a fluorescent group through a hydroxyl group. The concentration of the polystyrene microsphere was calculated as reported (Walton et al., 2001; Zhang et al., 2018).

### G-LISA

G-LISA activation assay was performed to determine GTP-bound Rho in cell lysates for evaluating the RhoA activity of PMVECs (Cytoskeleton, Denver, Colorado, United States). All operations follow the instructions (Aguilar et al., 2011).

### Proteomics Analysis

The LC-MS/MS analysis was carried out in CapitalBio Technology (Beijing, China). Briefly, at the end of the 10-day exhausted-exercise protocol, lung tissue protein was extracted and quantified using the BCA Protein Assay Kit (Thermo Fisher Scientific, Rockford, IL, United States). The protein was labeled by using TMT Mass Tagging Kits and Reagents (Pierce, Rockford, IL, United States) with different reporter ions (126–131 Da) for relative quantification. The labeled peptide aliquots were combined for fractionation and further LC-MS analysis by using a Q Exactive mass spectrometer (Thermo Fisher Scientific, Rockford, IL, United States). Twenty most intense precursor ions from a survey scan were selected for MS/MS detected at a mass resolution of 35,000 at m/z of 400 in an Orbitrap analyzer (Thermo Fisher Scientific, Rockford, IL, United States). All the tandem mass spectra were produced by the higher energy collision dissociation (HCD) method. The MS/MS data were collected and searched against the Rattus norvegicus database using the SEQUEST algorithms. A protein with fold change ≥ 1.5 and the presence of at least two unique peptides with a *p*-value < 0.05 was considered to be a differentially expressed protein (DEP). At last, DEPs were analyzed by referring to three key databases: Gene Ontology (GO), Kyoto Encyclopedia of Genes and Genomes (KEGG), and Reactome and the Clusters of Orthologous Groups (COG) (Xu et al., 2019). The mass spectrometry proteomics data have been deposited to the ProteomeXchange Consortium via the PRIDE (Deutsch et al., 2020) partner repository with the dataset identifier PXD032737.

### Transendothelial Permeability Assay

Transendothelial permeability was measured as described (Shi et al., 2016). The transwell filters (0.4 μm pore size, 12 mm in diameter, Corning, MA, United States) were coated with gelatin (0.1%, Merck Millipore, Darmstadt, Germany). PMVECs were seeded onto the membrane at a density of 2 × 10^5^ cells/well for 3 days to attain confluence. FITC-dextran (molecular weight 4,200; Sigma) was added to the upper chamber at a concentration of 1 mg/ml, and the cells were incubated in normoxic or hypoxic conditions as described previously. A sample of 200 μl from the lower chamber was taken and fluorescence intensity was measured with a microplate reader at an excitation of 490 nm and emission of 520 nm.

### Determination of mRNA Expression Levels via Real-Time PCR

The mRNA levels of Mypt1 were analyzed by RT-PCR. The total RNA of PMVECs was extracted by using the TRIpure Reagent (DiNing, Beijing, China) according to the manufacturer’s instructions with RNase-free conditions. The total RNA (2 μg) was reversely transcribed to cDNA using an Integrated First-strand cDNA Synthesis Kit with a gDNA Eraser (DiNing, Beijing, China) according to the instructions. The specific transcripts were assessed by quantitative RT-PCR using a 2x Fast qPCR Master Mixture (DiNing, Beijing, China) and analyzed with the Agilent AriaMx RT-PCR system (Agilent, CA, United States). Gene-specific primers were synthesized by Tsingke Biological Technology (Beijing, China) and the following primer sequences were used: CTA​CGA​GAA​CCA​GAG​AAC​AAG​AA (Forward) and GCA​TAG​AGT​GAA​CTG​CCT​AGA​G (Reverse) for Mypt1, ATC​ATT​GCT​CCT​CCT​GAG​CG (Forward) and GCA​TAG​AGT​GAA​CTG​CCT​AGA​G (Reverse) for β-actin. The mRNA levels were normalized to β-actin mRNA levels. PCR was conducted at 95°C for 1 min, 40 cycles at 95°C for 10 s, and at 60°C for 30 s. Relative mRNA expression was calculated by the comparative CT method.

### Western Blot

Rat lung tissues were removed after modeling and immediately frozen at -80°C till use. PMVECs were collected following incubation sequentially with control or YPF rat serum in normoxic or hypoxic conditions. Lung tissue and PMVECs were homogenized in a lysis buffer containing the protease and phosphatase inhibitor. The protein concentration was determined by the bicinchoninic acid assay method. The protein was separated by sodium dodecyl sulfate-polyacrylamide gel electrophoresis and transferred to the polyvinylidene fluoride (PVDF) membrane. After blocking non-specific binding sites with 3% bovine serum albumin or 3% skimmed milk powder in Tris-buffered saline Tween (TBST), the PVDF membranes were incubated overnight at 4°C with primary antibodies against p-MLC (1:1,000), MLC (1:1,000), RhoA (1:1,000), Rock (1:1,000), p-Mypt1 (1:1,000), Mypt1 (1:1,000), Gng12 (1:800), p-PAK1 (1:1,000), PAK1(1:1,000), p-MLCK (1:1,000), MLCK (1:1,000), claudin 3 (1:1,000), claudin 18 (1:1,000), claudin 5 (1:1,000), VE-cadherin (1:1,000), occludin (1:1,000), β-catenin (1:1,000), siah2 (1:1,000), and β-actin (1:2000). Following rinsing with TBST three times, the PVDF membranes were incubated with secondary antibodies (EMAR, Beijing, China) at room temperature for 1 h, and antibody binding was detected by using chemiluminescence detection kit (Applygen, Beijing, China). Bands were scanned by Bio-Rad Quantity One software (Bio-Rad, Richmond, CA, United States), while band intensities were quantified by Image J software (Bethesda, MD, United States).

### Statistical Analysis

All parameters were expressed as means ± SEM. Statistical analysis was performed by one-way ANOVA followed by Tukey’s test for multiple comparisons or using two-way ANOVA followed by Tukey’s test for multiple comparisons. Data were analyzed using GraphPad Prism 7 software (GraphPad software Inc., United States). *p* < 0.05 was considered to be statistically significant.

## Results

### Yu-Ping-Feng Prevents Reduction in Food-Intake Weight/Body Weight and Improves Changes in Lung Manifestation Induced by Exhausted Exercise

**FIGURE 1:**
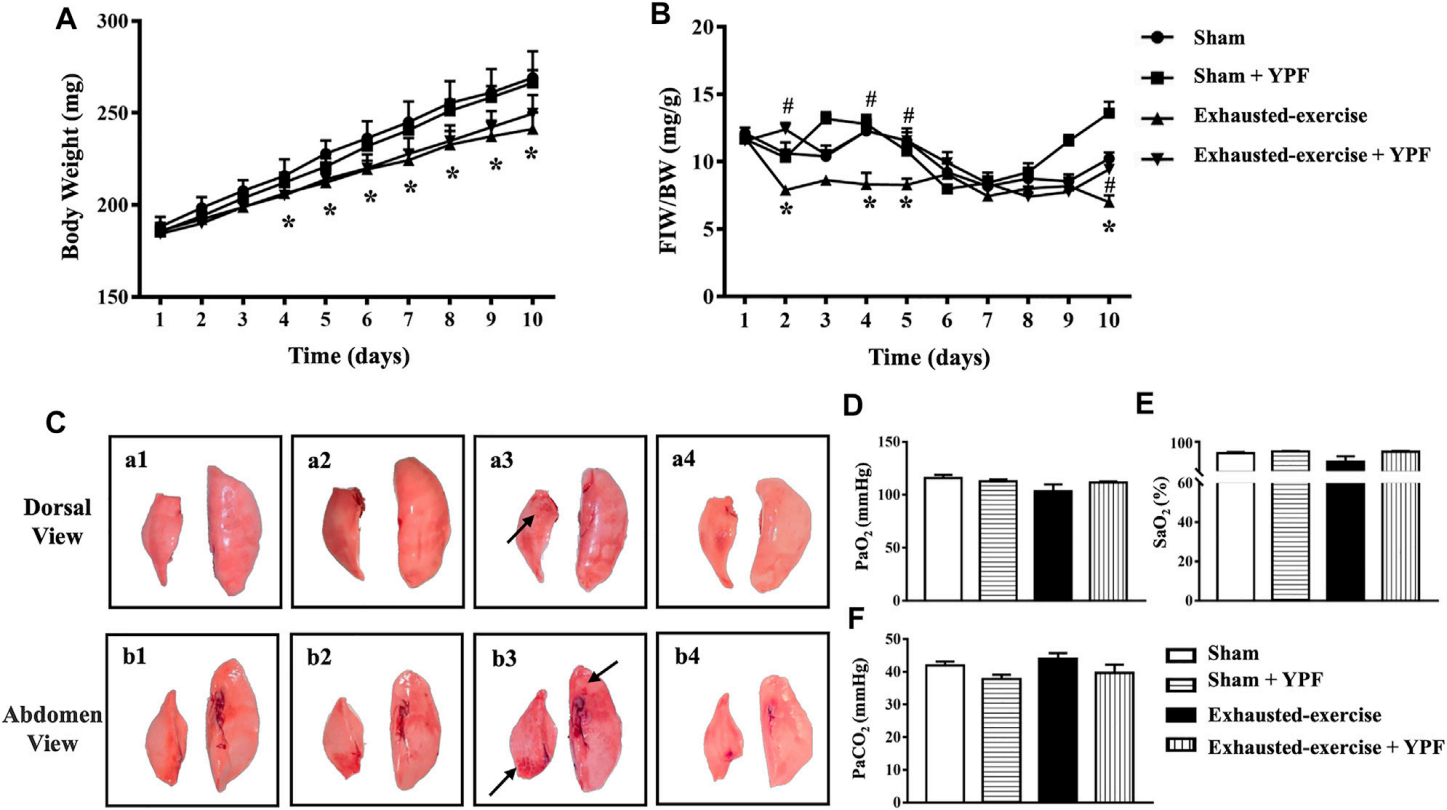
YPF prevents the reduction in food-intake weight (FIW)/body weight (BW) and improves changes in the lung macro morphology induced by exhausted exercise. **(A)**Alteration of BW with time in four groups. **(B)** Change in FIW/BW with time in four groups. Values are means ± SEM from eight animals. * *p* < 0.05 vs. sham; #*p* < 0.05 vs. exhausted-exercise. **(C)** Representative images of lung macro morphology in four groups. Arrows: swollen and hemorrhage area. **(a1, b1)** Sham group; **(a2, b2)** sham + YPF group; **(a3, b3)** exhausted-exercise group; and **(a4, b4)** exhausted-exercise + YPF group. **(D)** Partial arterial pressure of oxygen. **(E)** Saturation of arterial blood oxygen. **(F)** Partial arterial pressure of carbon dioxide. Results are presented as means ± SEM (*n* = 6).

The change in body weight (BW) and FIW/BW in different groups with time are shown in Figures 1A,B. As noticed, exhausted exercise caused a decrease in both BW and FIW/BW. YPF had no effect on the decrease in BW after exhausted exercise for 10 days (Figure 1A), while it attenuated the decreases in FIW/BW (Figure 1B).

Macroscopically, the left lobe and the right middle lobe lungs of animals from the exhausted-exercise group became deep pink, swollen, and hemorrhaged (Figures 1C, a3,b3, arrows) compared with the lungs of animals from the sham (Figure 1C, a1 and b1) and sham + YPF groups (Figures 1C, a2,b2). Treatment with YPF abolished the impairments in the lungs after exhausted exercise obviously (Figures 1C, a4,b4).

Shown in Figures 1D–F is the arterial blood gas analysis in terms of PaO_2_, SaO_2_, and PaCO_2_ with no difference observed among groups.

### Yu-Ping-Feng Improves Exhausted-Exercise-Induced Lung Edema and Lung Injury

**FIGURE 2:**
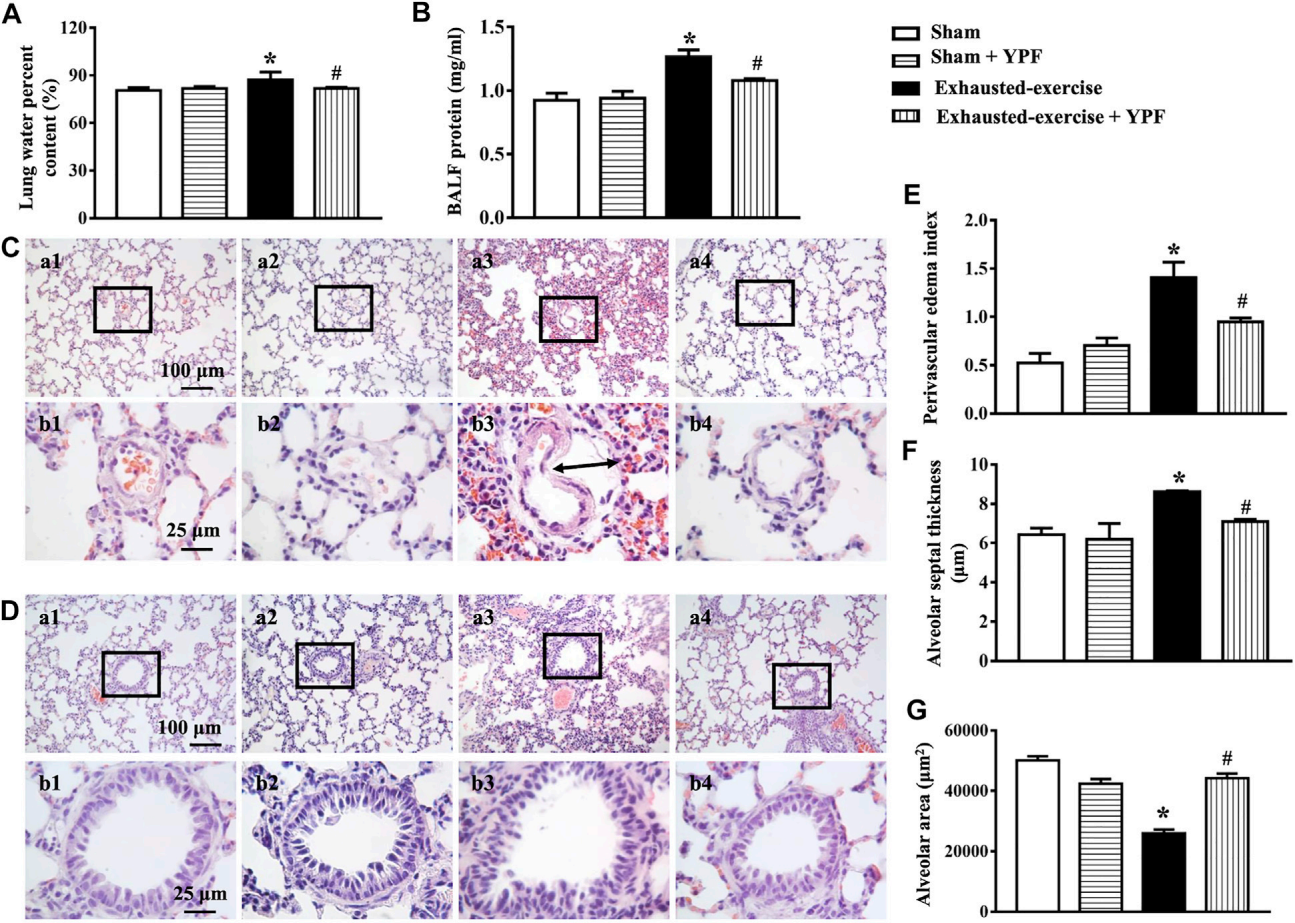
YPF attenuates lung edema and the morphological alteration induced by exhausted exercise. **(A)** Lung water percent content in different groups. **(B)** BALF protein concentration in different groups. **(C)** Representative H&E staining images of rat lung microvessels, alveolar space, and alveolar septum. **(D)** Representative H&E staining images of rat terminal bronchioles. **(a1, b1)** Sham group; **(a2, b2)** sham + YPF group; **(a3, b3)** exhausted-exercise group; and **(a4, b4)** exhausted-exercise + YPF group. Upper scale bar = 100 μM, the amplified multiple is 100×; lower scale bar = 25 μM, the amplified multiple is 400×. The arrow indicates perivascular edema. **(E)** Perivascular edema index. **(F)** Quantitative data for alveolar septal thickness. **(G)** Quantitative data for the alveolar area. Results are presented as means ± SEM (*n* = 8 in A and B, and *n* = 4 in E–G. * *p* < 0.05 vs. sham; #*p* < 0.05 vs. exhausted-exercise.

Lung edema caused by exhausted exercise was determined by the lung water percent content. In the exhausted-exercise group, the lung water percent content increased, which was counteracted by YPF treatment (Figure 2A). In addition, the protein level in BALF was used to evaluate alveolar-capillary membrane permeability. Exhausted exercise distinctly increased protein concentration in rat BALF, which was inhibited by YPF treatment (Figure 2B).

Exhausted exercise led to apparent alterations in lung histology, manifesting the thickening of the alveolar septa, congestion of the alveolar space, abnormal arrangement of the trachea epithelium, and perivascular edema (Figures 2C,D, a3,b3), which were alleviated by treatment with YPF obviously (Figures 2C,D, a4,b4). The statistical results of H&E images shown in Figures 2E–G confirmed the results from the survey.

### Yu-Ping-Feng Protects the Integrity of Cell Junctions of the Alveolar-Capillary Barrier

**FIGURE 3:**
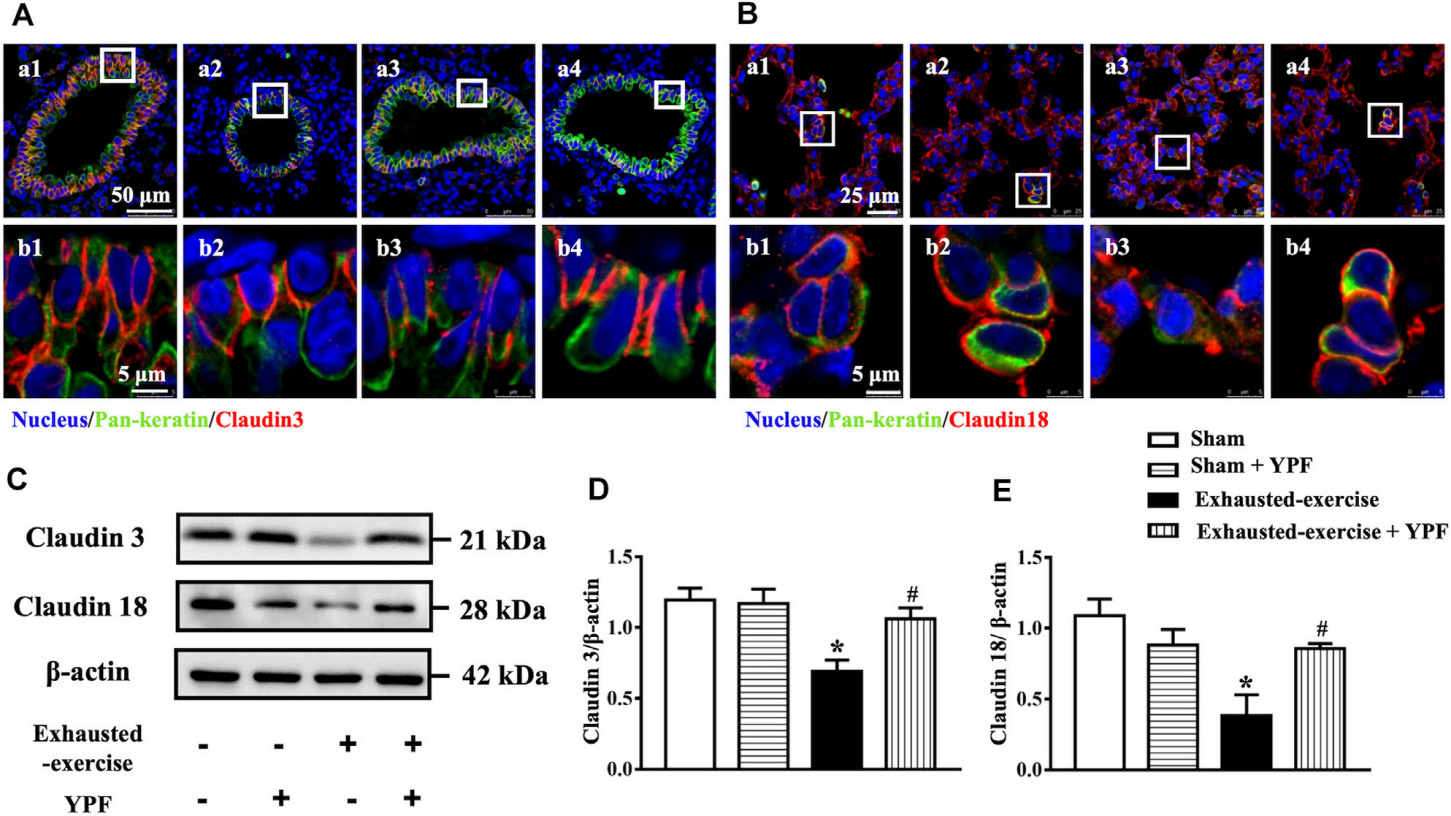
YPF maintains the integrity of cell junctions in the pulmonary epithelium. **(A)** Representative immunofluorescent staining images of claudin 3 in the lung tracheal epithelium. Claudin 3 (red) localized between the epithelial cells with marker E-cadherin (green). Nuclei stained blue. **(a1, b1)** Sham group; **(a2, b2)** sham + YPF group; **(a3, b3)** exhausted-exercise group; and **(a4, b4)** exhausted-exercise + YPF group. Upper scale bar = 50 μM; lower scale bar = 5 μM. **(B)**Representative immunofluorescent staining images of claudin 18 in the alveolar epithelium. Claudin 18 (red) localized between the epithelial cells with marker E-cadherin (green) and nuclei stained blue. **(a1, b1)** Sham group; **(a2, b2)** sham + YPF group; **(a3, b3)** exhausted-exercise group; and **(a4, b4)** exhausted-exercise + YPF group. Upper scale bar = 25 μM; lower scale bar = 5 μM. **(C)**Representative Western bands of claudin 3 and claudin 18. β-Actin was used as a loading control; *n* = 4. **(D–E)** Depicted in D and E are the semiquantitative analyses of claudin 3 and claudin 18, respectively. Results are presented as means ± SEM (*n* = 4). * *p* < 0.05 vs. sham; #*p* < 0.05 vs. exhausted-exercise.

Epithelial and endothelial cell junctions play a critical role in the integrity of the alveolar-capillary barrier in the lungs (Herrero et al., 2018). Given the lung edema after exhausted exercise in rats, we determined the tight junctions and adherens junctions of rat lung tissue. First, we observed the morphology of the tight junctions of the lung epithelium by immunofluorescence. The results revealed that exhausted exercise caused a discontinuity of claudin 3 and claudin 18 staining around the trachea epithelium (Figures 3A, a3,b3) and alveolar cells (Figures 3B, a3,b3), respectively. The exhausted exercise caused-discontinuity of claudins was protected by treatment with YPF (Figures 3A,B, a4,b4). Western blot showed that the expression of both claudin 3 and claudin 18 was significantly downregulated by exhausted exercise, which was prevented by YPF treatment (Figures 3C–E).

**FIGURE 4:**
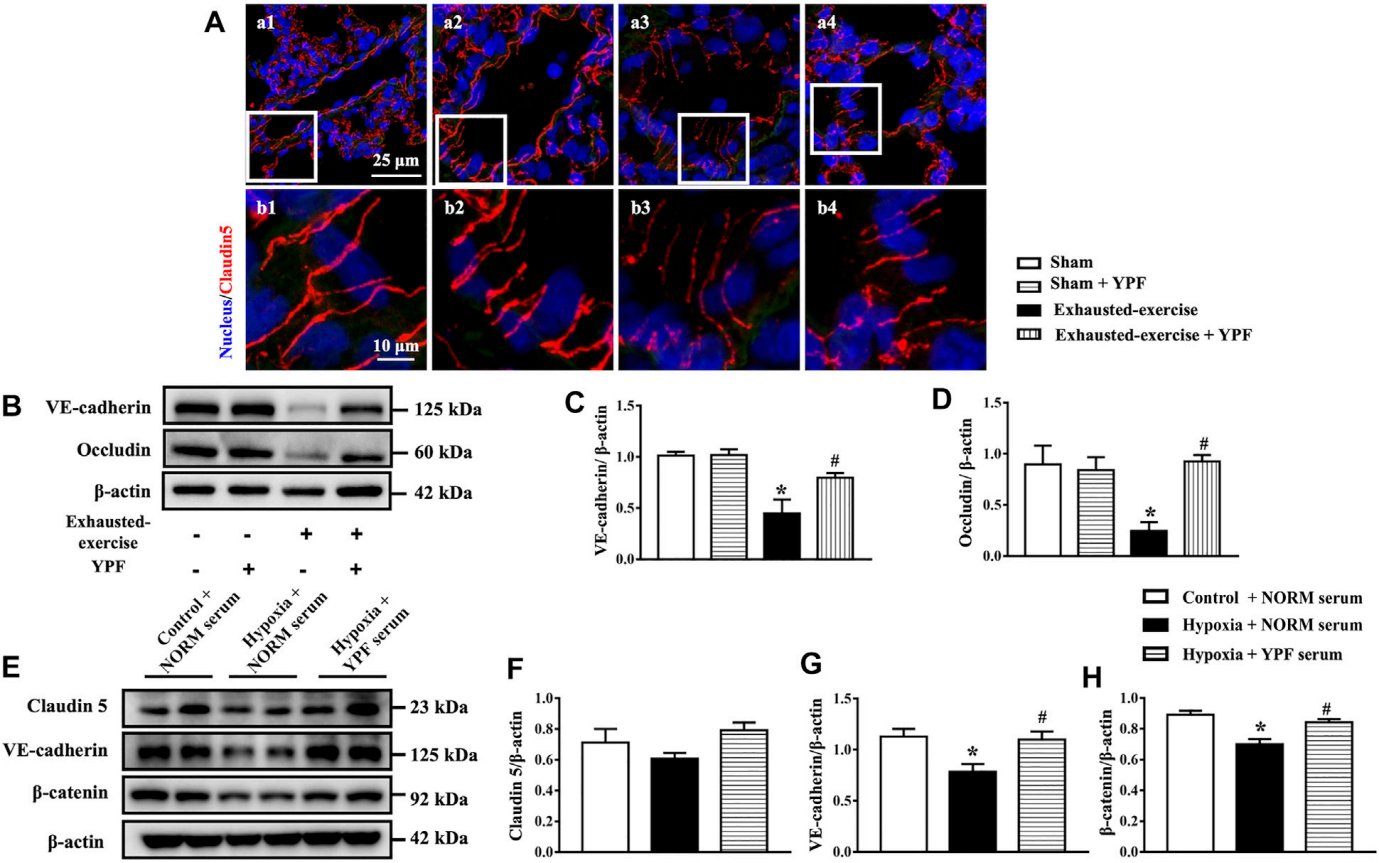
YPF maintains the integrity of cell junctions in the pulmonary endothelium and the effect of YPF serum on pulmonary microvascular cells. **(A)**Representative immunofluorescent staining images of claudin 5 in lung microvascular. Claudin 5 stained red and nuclei stained blue. **(a1, b1)** Sham group; **(a2, b2)** sham + YPF group; **(a3, b3)** exhausted-exercise group; and **(a4, b4)** exhausted-exercise + YPF group. Upper scale bar = 25 μM; lower scale bar = 10 μM. **(B)**Representative Western bands of VE-cadherin and occludin in lung tissue. **(C)** and **(D)** Semi-quantitative analysis of VE-cadherin and occludin. Results are presented as means ± SEM (*n* = 4). **(E)** Representative Western bands of claudin 5, VE-cadherin, and β-catenin in PMVECs. **(F–H)**Semi-quantitative analysis of claudin 5, VE-cadherin, and β-catenin. Results are presented as means ± SEM (*n* = 6). * *p* < 0.05 vs. control + NORM serum; #*p* < 0.05 vs. hypoxia + NORM serum.

Immunofluorescence staining of claudin 5 was conducted for pulmonary microvessels. Similarly, exhausted exercise led to lighter staining and discontinuity of claudin 5 (Figure 4A, a3,b3), indicating a downregulated expression of the junction protein. The exhausted exercise-impaired staining of claudin 5 was obviously improved by treatment with YPF (Figure 4A, a4,b4).

In addition, the expression of occludin, a tight junction protein, and VE-cadherin, an adherens junction protein, was assessed by Western blot as well, revealing a decreased expression of both proteins after exhausted exercise. These changes were attenuated by YPF (Figures 4B–D).

Taken together, YPF prevented the downregulated expression of tight and adherens junction proteins after exhausted exercise, maintaining the integrity of the epithelium and endothelium barrier, thus refraining from lung edema.

### Yu-Ping-Feng Serum Ameliorates Hyperpermeability of Pulmonary Microvascular Endothelial Cells Induced by Hypoxia

The potential of YPF to prevent hyperpermeability of the endothelial cell barrier was documented *in vitro* by testing the effect of YPF serum on hypoxia-challenged PMVECs. Many chemical constituents were effectively separated from the YPF solution and YPF serum through HPLC/Q-TOF-MS. Astragaloside Ⅳ, calycosin, formononetin, atractylenolide Ⅰ, atractylenolide Ⅱ, prim-O-glucosylcimifugin, and cimifugin were used as standards according to the Chinese Pharmacopeia 2020 Edition. The results suggested that YPF serum did not only contain the aforementioned seven components but also contains 178 other components that were not found in the YPF solution according to the Thermo Compound Discovery database (Supplementary Figure S1; Supplementary Table S1). These metabolites have interesting effects including anti-inflammatory, anti-oxidation, anti-tumor, protecting endothelial cells, and other effects (Zhang et al., 2012; Cheng and Wei, 2014; Guo et al., 2017; Jiang et al., 2019; Zhou et al., 2020).

Then, the optimal concentration of YPF serum was first assessed by cell viability assays. The results showed that 6.25% YPF serum and 12.5% YPF serum had no obvious cytotoxicity (Supplementary Figure S2A). In the study of transendothelial permeability, 6.25% YPF serum had a trend to protect the hyperpermeability of PMVECs after hypoxia but without statistical significance (Supplementary Figure S2B), while 12.5% YPF serum obviously decreased the hyperpermeability of PMVECs induced by hypoxia (Supplementary Figure S2B). Thus, 12.5% YPF serum was used for further experiments. In addition, we examined the expression of cell junction proteins by Western blot *in vitro*. As shown in Figures 4E–H, hypoxia induced a decrease of claudin 5, VE-cadherin, and β-catenin, while YPF improved this alteration significantly.

### Proteomics Analysis Identified the Differentially Expressed Proteins by TMT Labeling

TMT labeling quantitative proteomics was performed to detect the alteration of protein expression among the sham group, exhausted-exercise group, and exhausted-exercise + YPF group. A total of 7,158 valid proteins were successfully identified with at least one unique peptide and less than a 1% false discovery rate (FDR). Proteins with a fold-change of 1.5 and with a significant *p*-value (*p* < 0.05) were regarded as differentially expressed proteins (DEPs) between the two groups. There were 165 DEPs and 175 DEPs identified between the exhausted-exercise group and the sham group, and between the exhausted-exercise + YPF group and the exhausted-exercise group, respectively. The significant enrichment GO terms and pathway (top 30) between the exhausted-exercise group and the sham group are shown in Supplementary Figure S3. Similarly, Supplementary Figure S4, S5 shows the enrichment GO terms and pathways in the exhausted-exercise + YPF group and the exhausted-exercise group. All the enriched pathways are related to the regulation of actin cytoskeleton, GCPR ligand binding, and energy metabolism.

**FIGURE 5:**
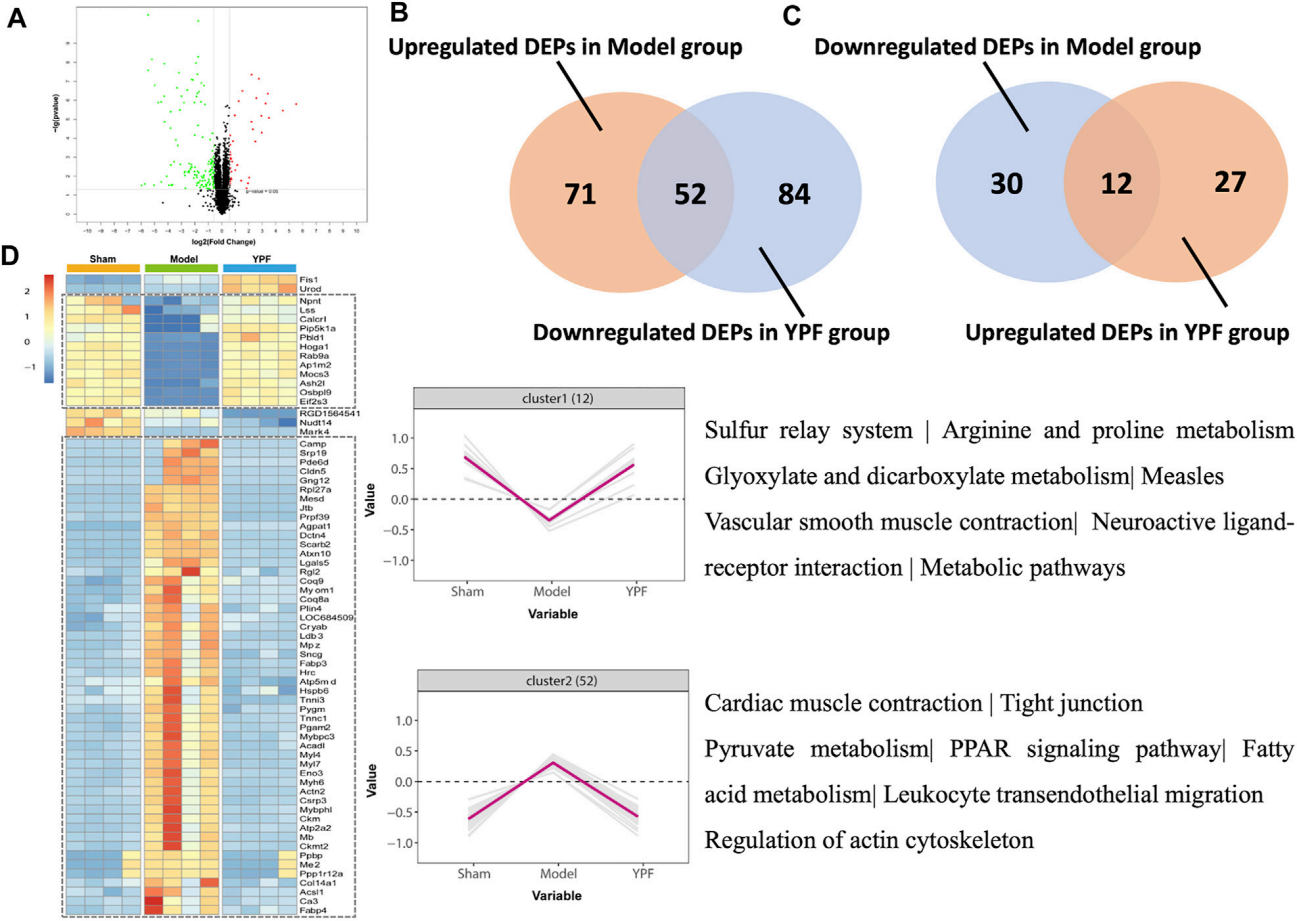
Quantitative proteomic study on rat lung tissue. **(A)**Volcano plot of differentially expressed proteins (DEPs) identified between the exhausted-exercise group and the exhausted-exercise + YPF group. Red dots: represent upregulated DEPs; green dots: represent downregulated DEPs; and black dots: represent unchanged proteins. **(B)** Venn diagram of significantly changed proteins in upregulated DEPs of the exhausted-exercise group and downregulated DEPs of the exhausted-exercise group + YPF group with 52 proteins in intersection area. **(C)** Venn diagram of significantly changed proteins in downregulated DEPs of the exhausted-exercise group and upregulated DEPs of the exhausted-exercise group + YPF group with 12 proteins in the intersection area. **(D)** Heatmap of adjusting DEPs by YPF. The color bar represents the fold change from increasing to decreasing of all proteins identified in each group. Hierarchical clusters show a clear group differentiation according to similarity. Numbers of proteins and selected enriched KEGG pathways are indicated for marked clusters.

Interestingly, we found that 123 proteins were upregulated, and 42 proteins were downregulated between the exhausted-exercise group and sham group (Supplementary Figure S6A) and a total of 39 proteins were upregulated, and 136 proteins were downregulated adversely between the exhausted-exercise + YPF group and exhausted-exercise group (Figure 5A) with 69 overlapping proteins between the two pairs (Supplementary Figure S6B). Between upregulated DEPs in the exhausted-exercise group and downregulated DEPs in the exhausted-exercise + YPF group, there were 52 overlapped proteins (Figure 5B). Similarly, between downregulated DEPs in the exhausted-exercise group and upregulated DEPs in the exhausted-exercise + YPF group, there were 12 overlapped proteins (Figure 5C). The heatmap was drawn based on the expression level of 69 overlapped proteins among the sham, exhausted-exercise, and exhausted-exercise + YPF groups (Figure 5D). Cluster 1 showed the high-low-high trend of proteins, while cluster 2 showed the low-high-low trend of proteins. The KEGG pathway enrichment analysis using DAVID 6.8 showed that the 69 overlapping proteins are involved in the regulation of actin cytoskeleton, metabolic pathways, and tight junction, among others. It is noteworthy that the regulation of actin cytoskeleton pathway was implicated in the protective effect of YPF on alveolar-capillary barrier injury, a finding that has not been reported previously, which impelled us to study the underlying mechanisms of YPF by focusing on the actin cytoskeleton.

### Yu-Ping-Feng Attenuates Exhausted-Exercise-Induced Alterations of Proteins in the Regulation of Actin Cytoskeleton *in Vivo*

There are the five proteins, Myl7, Ppp1r12a, Actin2, Pip5k1a, and Gng12 that were enriched in the pathway of the regulation of actin cytoskeleton. PP1r12a, also known as Mypt1, is a large myosin-binding submit of myosin phosphatase (MP) (Kiss et al., 2019). As the gamma 12 subunit of heterotrimeric G-proteins, Gng12, another differentially expressed protein, is also involved in the regulation of actin cytoskeleton. As shown in Supplementary Figure S7, all these proteins are involved in regulating the phosphorylation of MLC. Assessment by mass spectrometry found that Mypt1 and Gng12 were increased by exhausted exercise, which was relieved by YPF (Figure 5D).

**FIGURE 6:**
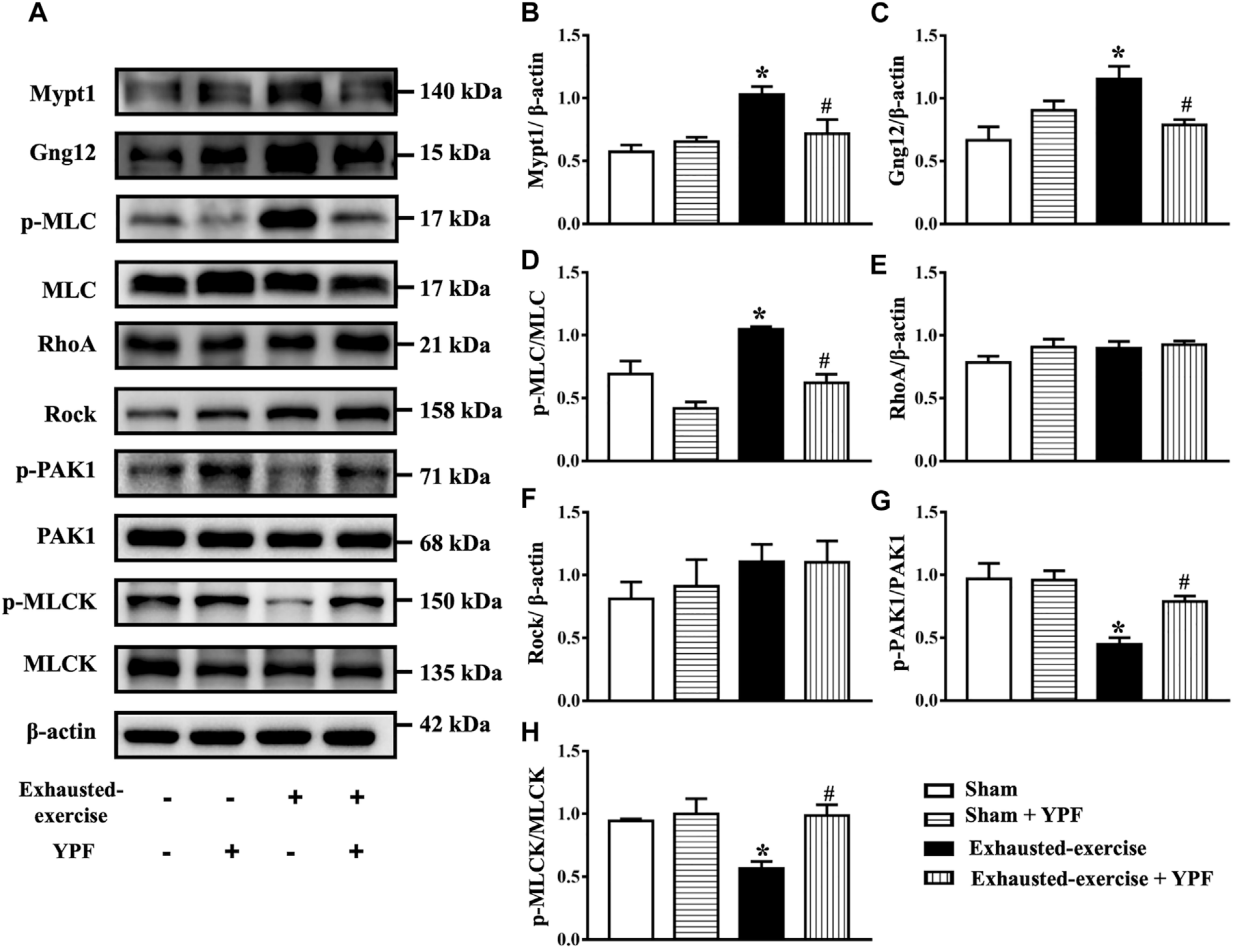
YPF attenuates the alteration of MLC and related signaling proteins after exhausted exercise. **(A)**Representative Western bands of Mypt1, Gng12, p-MLC, MLC, RhoA, Rock, p-PAK1, PAK1, p-MLCK, and MLCK. **(B–H)**Semi-quantitative analysis of **(B)** Mypt1, **(C)** Gng12 **(D)** p-MLC/MLC, **(E)** RhoA, **(F)** Rock, **(G)**, p-PAK1/PAK1, and **(H)** p-MLCK/MLCK . Results are presented as means ± SEM (*n* = 4). * *p* < 0.05 vs. sham; #*p* < 0.05 vs. exhausted-exercise.

Therefore, we first detected the expression level of Mypt1 and Gng12 *in vivo* by Western blot. As shown in Figures 6A–C, exhausted exercise induced an increase in the expression of Mypt1 and Gng12 while YPF inhibited this increase, in accordance with proteomics results. In addition, exhausted exercise induced an increase of p-MLC while YPF restored this alteration apparently (Figures 6A,D). On the other hand, the activation of RhoA increased after hypoxia (Supplementary Figure S8D), although the expression of RhoA and Rock had no significant changes among the four groups (Figures 6A,E,F). The phosphorylation level of PAK1 at the autophosphorylation site Ser 199 and the phosphorylation level of MLCK, the two downstream proteins of Gng12, were decreased by exhausted exercise, which were protected by YPF (Figures 6A,G,H), implying that YPF decreased the phosphorylation of MLC by acting at the two links.

### Yu-Ping-Feng Serum Activates the Siah2-Ubiquitin-Proteasomal Pathway to Degrade of Mypt1 and Gng12-PAK1-MLCK Thus Regulating Actin Cytoskeleton *in Vitro*

**FIGURE 7:**
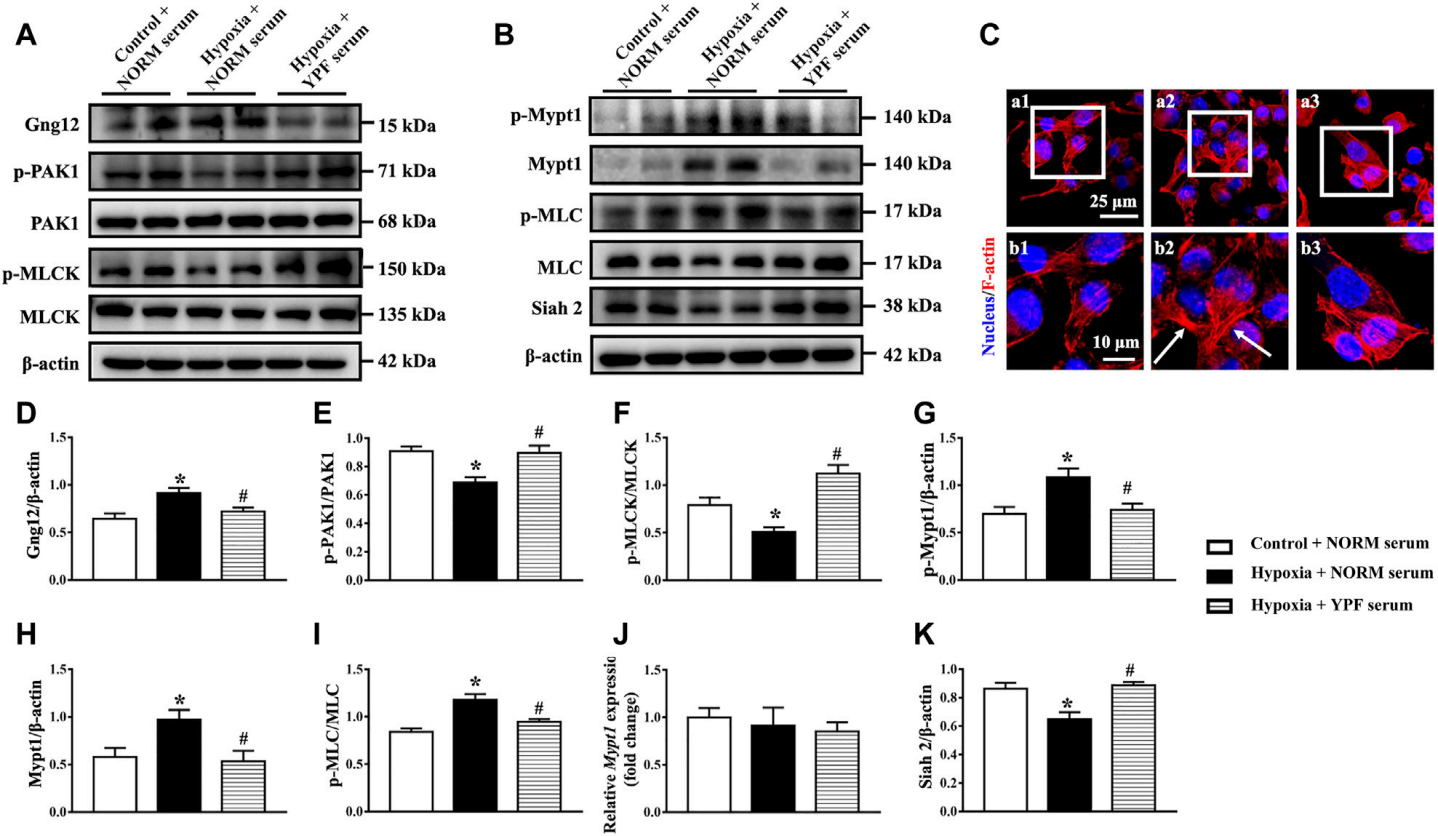
YPF serum attenuates the alteration of MLC and related signaling proteins in PMVECs. **(A)**Representative Western bands of Gng12, p-PAK1, PAK1, p-MLCK, and MLCK. **(B)**Representative Western bands of p-Mypt1, Mypt1, p-MLC, MLC, and Siah2. **(C)**Representative F-actin staining images (red) in PMVECs. Nuclei stained blue. Arrows: stress fibers. **(a1,b1)** Control + NORM serum; **(a2,b2)** hypoxia + NORM serum; and **(a3, b3)** Hypoxia + YPF serum. Upper scale bar = 25 μM; lower scale bar = 10 μM. **(D–I)**Semi-quantitative analysis of **(D)** Gng12, **(E)** p-PAK1/PAK1, **(F)** p-MLCK/MLCK, **(G)** p-Mypt1, **(H)** Mypt1, and **(I)** p-MLC/MLC . Results are presented as means ± SEM (n = 6). **(J)** mRNA expression of Mypt1; *n* = 8. **(K)** Semi-quantitative analysis of Siah2. Results are presented as means ± SEM (*n* = 6). * *p* < 0.05 vs. control + NORM serum; #*p* < 0.05 vs. hypoxia + NORM serum.

We further verified the role of YPF in regulating the cytoskeleton in PMVECs *in vitro*. The expression of RhoA and Rock remained unchanged among the groups, parallel with the results *in vivo* (Supplementary Figure S8A–C). However, the activity of RhoA increased in response to hypoxia, which was prevented by YPF serum (Supplementary Figure S8D). Similarly, the expression of Gng12 and downstream p-PAK1 and p-MLCK in PMVECs varied among groups in an identical manner as *in vivo* (Figures 7A,D–F). The results showed that the expression of p-Mypt1, Mypt1, and p-MLC was significantly increased by hypoxia compared with the control group, which was ameliorated by YPF serum (Figures 7B,G–I). To delve into the reason for Mypt1 variation in different groups, we first detected the transcriptional level of Mypt1 with no difference observed among all groups (Figure 7J). We next assessed the expression of Siah2, an E3-ligase known to interact with Mypt1 for the proteasomal degradation of Mypt1 (Twomey et al., 2010), while YPF serum counteracted this change (Figures 7B,K).

Finally, F-actin staining revealed that hypoxia caused F-actin remodeling, resulting in stress fiber formation. YPF serum pretreatment prevented hypoxia-induced changes in the actin cytoskeleton (Figure 7C).

### Yu-Ping-Feng Protects the Integrity of the Alveolar-Capillary Barrier After Exhausted Exercise

**FIGURE 8:**
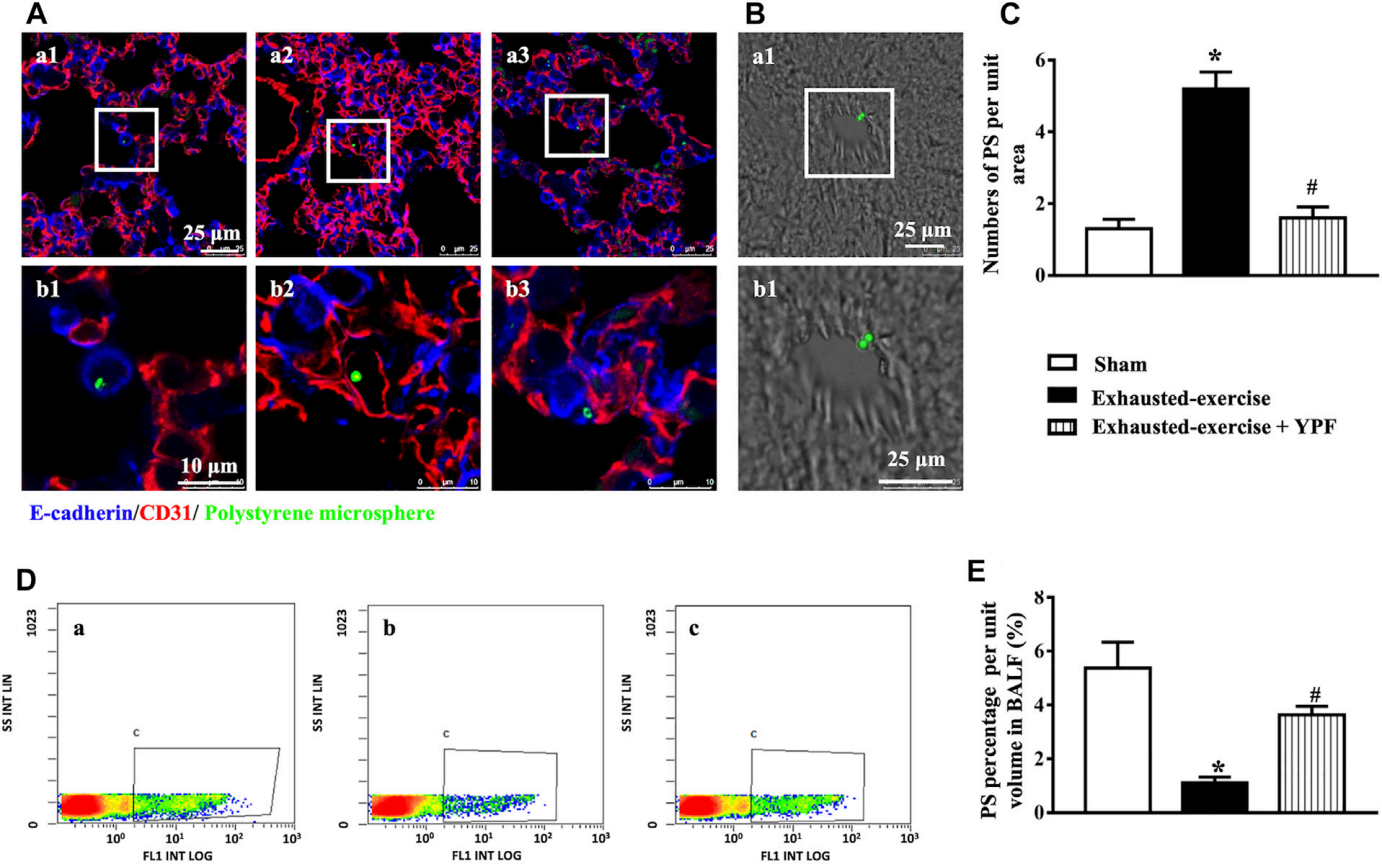
Integrity of the alveolar-capillary barrier evaluated by intratracheal instillation of the polystyrene microsphere. **(A)**Representative immunofluorescent staining images of lung tissue. The microvessel was marked by CD31 as red and alveoli were marked by E-cadherin as blue. Green dots represent the polystyrene microsphere. **(a1, b1)** Sham group; **(a2, b2)** exhausted-exercise group; and **(a3, b3)** exhausted-exercise + YPF group. Upper scale bar = 25 μM; lower scale bar = 10 μM. **(B)** Light field of liver tissue. Green dots represent the polystyrene microsphere. **(a1, b1)** Exhausted-exercise group. Scale bar = 25 μM. **(C)**Number of polystyrene microspheres per unit area in liver tissue sections. Results are presented as means ± SEM (*n* = 3). **(D)** Representative image of flow cytometry. **(a)** Sham group; **(b)** exhausted-exercise group; and **(c)** exhausted-exercise + YPF group. **(E)**Percentage of polystyrene microspheres per volume in BALF. Results are presented as means ± SEM (*n* = 4). * *p* < 0.05 vs. sham; #*p* < 0.05 vs. exhausted-exercise.

Following intratracheal instillation of the polystyrene microsphere, lung sections were observed by immunofluorescent staining. Results showed that more polystyrene microspheres appeared in pulmonary microvessels in the exhausted-exercise group (Figure 8A, a2,b2), while more polystyrene microspheres appeared in the alveolar space in the sham and exhausted-exercise + YPF groups (Figure 8A, a1,b1, a3,b3). Considering that the polystyrene microspheres cross the pulmonary alveolar-capillary barrier entering the circulating blood (West and Mathieu-Costello, 1999), we also observed liver sections by the light field of a laser scanning confocal microscope (Figure 8B). As expected, more polystyrene microspheres were observed in the exhausted-exercise group, compared with the sham and YPF treatment groups (Figure 8C). Then we analyzed the polystyrene microspheres in BALF by flow cytometry (Figures 8D,E), revealing that exhausted exercise decreased polystyrene microspheres in BALF, which was prevented by YPF.

## Discussion

The exhausted-exercise-caused alveolar-capillary barrier dysfunction has been noticed. A study found that intense exercise increased concentrations of red cells and proteins in the bronchoalveolar lavage fluid (BALF) from athletes by altering the structure and function of the alveolar-capillary barrier (Hopkins et al., 1997). It is reported that rats subjected to exhaustive exercise presented a decrease in the tight junction protein occludin and impairing the alveolar-capillary barrier leading to the leakage of proteins into the alveolar space (Zhou et al., 2017). Consistent with these results, the present study demonstrated that exhausted-exercise induced cell junction disassembly leading to alveolar-capillary-barrier injury, which is evidenced not only by the finding in the macro and microscopic morphology of lung tissue, but also by the intratracheal instillation of polystyrene microspheres, which confirmed the dysregulation of the alveolar epithelial barrier function. Noticeably, the *in vitro* study found that exhausted-exercise-caused insult in alveolar-capillary-barrier injury was at least partially ascribed to stress fiber formation. Treatment with YPF protected the stress fiber formation alongside restoration of the alveolar-capillary barrier, suggesting YPF as an option for the treatment of lung edema.

The alveolar-capillary barrier consists of the alveolar epithelium, the capillary endothelium, and the intervening extracellular matrix (West and Mathieu-Costello, 1999) with tight and adherens junctions between endothelial and epithelial cells as a major mediator (Herrero et al., 2018). The alveolar-capillary barrier function depends not only on the expression of junction proteins but also the cytoskeleton, which connects to junction proteins via adapter proteins (Thiery et al., 2009; Wettschureck et al., 2019).

Stress fibers are highly organized contractable cytoskeletons occurring in motile and non-motile cells. In vascular endothelial cells, stress fiber formation evokes a change in cell shape resulting in the breakdown of the endothelial cell barrier. It is, thus, most likely that the present study observed a protective effect of YPF on the stress fiber formation which underlies its role in attenuating alveolar-capillary barrier injury after exhausted exercise.

Stress fibers consist of many proteins including myosin light chain (MLC), the phosphorylation of which promotes actomyosin cross-bridge formation and contraction. MLC phosphorylation is governed by the balance of MLCK and MLC phosphatase with the former enhancing whereas the latter fading the phosphorylation of MLC. The activity of MLCK and MLC phosphatase is tightly controlled by a spectrum of signaling. To this end, the phosphorylation of MLCK by activated PAK1 is known to reduce the activity of MLCK (Sanders et al., 1999; Murthy et al., 2003). PAK is a p21-activiated kinase, which is reported to be inhibited by G-protein βγ subunits in mammals (Wang et al., 1999). G protein, as a second messenger, consists of *α*, *β*, and *γ* subunits (Asano et al., 1998) and transmits signals from an agonist-stimulate receptor to various downstream effectors (Neer, 1995). The present study revealed that hypoxia caused an increase in Gng12, γ subunit of G-proteins, a decrease in p-PAK, and a decrease in p-MLCK, consistent with the well-recognized reaction cascade that mediates the activity of MLCK. The decrease in p-MLCK and the resultant increase in the activity of MLCK contribute partly to the increase in p-MLC observed *in vivo* and *in vitro*. On the other hand, MLC phosphatase is a trimeric complex including a 110–133 kDa myosin-targeting subunit 1 (Mypt1). The evidence available shows that the activity of MLC phosphatase is negatively regulated by Mypt1 phosphorylation (Kiss et al., 2019). RhoA/Rock is known to increase the phosphorylation of MLC directly following RhoA activation (Pan et al., 2021), and is also reported to mediate Mypt1 phosphorylation (Kimura et al., 1996). In the present study, exhausted exercise and hypoxia did not increase the expression of RhoA and Rock, but increased RhoA activity. In addition, the expression of Mypt1 was elevated after both exhausted exercise and hypoxia, which is likely to boost the downregulation of MLC phosphatase, albeit the transcriptional level of Mypt1 did not change. Given the reported role of Siah2, an E3-ligase in the proteasomal degradation of Mypt1 in mammalian cells (Twomey et al., 2010), we examined the expression of Siah2 *in vitro*, revealing a decrease in the expression of Siah2 after hypoxia, which explained why the expression of Mypt1 increased in this circumstance, implying the involvement of the proteasome in the downregulation of MLC phosphatase and increase in MLC phosphorylation.

The results from the present study indicate that exhausted exercise/hypoxia-caused stress fiber formation is a scenario implicating a range of signaling, such as Gng12/PAK, RhoA/ROCK, and proteasome. Importantly, YPF restored all the impairments after exhausted exercise or hypoxia, proving the multi-targeting potential of YPF. Nevertheless, the detailed mechanism of the YPF effect requires further clarification and much more work is needed to identify the components in YPF responsible for the effects observed.

## Conclusion

The result of the present study provides evidence of the ability of YPF to tackle alveolar-capillary barrier injury, and the mechanism behind the beneficial role of YPF implicates the prevention of stress fiber formation in vascular endothelial cells and epithelial cells. This result offers a novel insight for a better understanding of the role of YPF in the fight against respiratory diseases, and highlights YPF as management for patients subjected to exhausted-exercise challenges or at risk of alveolar-capillary barrier injury.

## Data Availability

The datasets presented in this study can be found in online repositories. The names of the repository/repositories and accession number(s) can be found below: Proteomics data are available *via* ProteomeXchange with identifier PXD032737.

## References

[B1] Aguilar H. N. Tracey C. N. Tsang S. C. McGinnis J. M. Mitchell B. F. (2011). Phos-Tag-Based Analysis of Myosin Regulatory Light Chain Phosphorylation in Human Uterine Myocytes. PLoS One 6 (6), e20903. 10.1371/journal.pone.0020903 21695279 PMC3111472

[B2] Asano T. Morishita R. Ueda H. Asano M. Kato K. (1998). GTP-Binding Protein Gamma12 Subunit Phosphorylation by Protein Kinase C-Iidentification of the Phosphorylation Site and Factors Involved in Cultured Cells and Rat Tissues *In Vivo* . Eur. J. Biochem. 251 (1-2), 314–319. 10.1046/j.1432-1327.1998.2510314.x 9492299

[B3] Caillaud C. Serre-Cousiné O. Anselme F. Capdevilla X. Préfaut C. (1995). Computerized Tomography and Pulmonary Diffusing Capacity in Highly Trained Athletes after Performing a Triathlon. J. Appl. Physiol. 79 (4), 1226–1232. 10.1152/jappl.1995.79.4.1226 8567566

[B4] Cheng X. D. Wei M. G. (2014). Profiling the Metabolism of Astragaloside IV by Ultra Performance Liquid Chromatography Coupled with Quadrupole/Time-Of-Flight Mass Spectrometry. Molecules 19 (11), 18881–18896. 10.3390/molecules191118881 25407723 PMC6271624

[B5] Deutsch E. W. Bandeira N. Sharma V. Perez-Riverol Y. Carver J. J. Kundu D. J. (2020). The ProteomeXchange Consortium in 2020: Enabling 'Big Data' Approaches in Proteomics. Nucleic Acids Res. 48 (D1), D1145–D1152. 10.1093/nar/gkz984 31686107 PMC7145525

[B6] Fahlman M. M. Engels H. J. (2005). Mucosal IgA and URTI in American College Football Players: a Year Longitudinal Study. Med. Sci. Sports Exerc 37 (3), 374–380. 10.1249/01.mss.0000155432.67020.88 15741834

[B7] Flynn M. G. McFarlin B. K. (2006). Toll-Like Receptor 4: Link to the Anti-Inflammatory Effects of Exercise? Exerc Sport Sci. Rev. 34 (4), 176–181. 10.1249/01.jes.0000240027.22749.14 17031256

[B8] Gleeson M. Bishop N. C. Stensel D. J. Lindley M. R. Mastana S. S. Nimmo M. A. (2011). The Anti-Inflammatory Effects of Exercise: Mechanisms and Implications for the Prevention and Treatment of Disease. Nat. Rev. Immunol. 11 (9), 607–615. 10.1038/nri3041 21818123

[B9] Guo Y. Yin T. Wang X. Zhang F. Pan G. Lv H. (2017). Traditional Uses, Phytochemistry, Pharmacology and Toxicology of the Genus Cimicifuga: A Review. J. Ethnopharmacol. 209, 264–282. 10.1016/j.jep.2017.07.040 28826891

[B10] Herrero R. Sanchez G. Lorente J. A. (2018). New Insights into the Mechanisms of Pulmonary Edema in Acute Lung Injury. Ann. Transl. Med. 6 (2), 32. 10.21037/atm.2017.12.18 29430449 PMC5799138

[B11] Hopkins S. R. Schoene R. B. Henderson W. R. Spragg R. G. Martin T. R. West J. B. (1997). Intense Exercise Impairs the Integrity of the Pulmonary Blood-Gas Barrier in Elite Athletes. Am. J. Respir. Crit. Care Med. 155 (3), 1090–1094. 10.1164/ajrccm.155.3.9116992 9116992

[B12] Huang R. Cui Y. C. Wei X. H. Pan C. S. Li Q. He S. Y. (2019). A Novel Traditional Chinese Medicine Ameliorates Fatigue-Induced Cardiac Hypertrophy and Dysfunction via Regulation of Energy Metabolism. Am. J. Physiol. Heart Circ. Physiol. 316 (6), H1378–H1388. 10.1152/ajpheart.00731.2018 30951366

[B13] Huang R. Zhang Y. Zhang Y. Zhang L. Pei L. Shu G. (2020). Evaluation of the Synergetic Effect of Yupingfeng San and Flos Sophorae Immaturus Based on Free Radical Scavenging Capacity. Biomed. Pharmacother. 128, 110265. 10.1016/j.biopha.2020.110265 32425327 PMC7233259

[B14] Jiang Z. Peng C. Huang W. Wu B. Zhang D. Ouyang H. (2019). A High Throughput Three-step Ultra-performance Liquid Chromatography Tandem Mass Spectrometry Method to Study Metabolites of Atractylenolide-III. J. Chromatogr. Sci. 57 (2), 163–176. 10.1093/chromsci/bmy098 30496359

[B15] Kimura K. Ito M. Amano M. Chihara K. Fukata Y. Nakafuku M. (1996). Regulation of Myosin Phosphatase by Rho and Rho-Associated Kinase (Rho-Kinase). Science 273 (5272), 245–248. 10.1126/science.273.5272.245 8662509

[B16] Kiss A. Erdődi F. Lontay B. (2019). Myosin Phosphatase: Unexpected Functions of a Long-Known Enzyme. Biochim. Biophys. Acta Mol. Cell Res. 1866 (1), 2–15. 10.1016/j.bbamcr.2018.07.023 30076859

[B17] Liao C. Liu T. Zeng Z. Wang D. Tang G. Wang H. (2020). Efficacy and Safety of Modified Yupingfeng Formula in Treating Allergic Rhinitis: A Protocol for Systematic Review and Meta Analysis. Med. Baltim. 99 (51), e23698. 10.1097/MD.0000000000023698 PMC7748353 33371114

[B18] Ma J. Zheng J. Zhong N. Bai C. Wang H. Du J. (2018). Effects of YuPingFeng Granules on Acute Exacerbations of COPD: a Randomized, Placebo-Controlled Study. Int. J. Chron. Obstruct Pulmon Dis. 13, 3107–3114. 10.2147/COPD.S170555 30323581 PMC6174891

[B19] McKenzie D. C. O'Hare T. J. Mayo J. (2005). The Effect of Sustained Heavy Exercise on the Development of Pulmonary Edema in Trained Male Cyclists. Respir. Physiol. Neurobiol. 145 (2-3), 209–218. 10.1016/j.resp.2004.06.010 15705536

[B20] Murthy K. S. Zhou H. Grider J. R. Brautigan D. L. Eto M. Makhlouf G. M. (2003). Differential Signalling by Muscarinic Receptors in Smooth Muscle: M2-Mediated Inactivation of Myosin Light Chain Kinase via Gi3, Cdc42/Rac1 and P21-Activated Kinase 1 Pathway, and M3-Mediated MLC20 (20 kDa Regulatory Light Chain of Myosin II) Phosphorylation via Rho-Associated Kinase/myosin Phosphatase Targeting Subunit 1 and Protein Kinase C/CPI-17 Pathway. Biochem. J. 374 (Pt 1), 145–155. 10.1042/BJ20021274 12733988 PMC1223565

[B21] Neer E. J. (1995). Heterotrimeric G Proteins: Organizers of Transmembrane Signals. Cell 80 (2), 249–257. 10.1016/0092-8674(95)90407-7 7834744

[B22] Pan C. S. Yan L. Lin S. Q. He K. Cui Y. C. Liu Y. Y. (2021). QiShenYiQi Pills Attenuates Ischemia/Reperfusion-Induced Cardiac Microvascular Hyperpermeability Implicating Src/Caveolin-1 and RhoA/ROCK/MLC Signaling. Front. Physiol. 12, 753761. 10.3389/fphys.2021.753761 34975519 PMC8718710

[B23] Rigonato-Oliveira N. C. Mackenzie B. Bachi A. L. L. Oliveira-Junior M. C. Santos-Dias A. Brandao-Rangel M. A. R. (2018). Aerobic Exercise Inhibits Acute Lung Injury: from Mouse to Human Evidence Exercise Reduced Lung Injury Markers in Mouse and in Cells. Exerc Immunol. Rev. 24, 36–44. 29461970

[B24] Samano M. N. Pazetti R. Prado C. M. Tibério I. C. Saldiva P. H. Moreira L. F. (2009). Effects of Pneumonectomy on Nitric Oxide Synthase Expression and Perivascular Edema in the Remaining Lung of Rats. Braz J. Med. Biol. Res. 42 (11), 1113–1118. 10.1590/S0100-879X2009001100019 19855908

[B25] Sanders L. C. Matsumura F. Bokoch G. M. de Lanerolle P. (1999). Inhibition of Myosin Light Chain Kinase by P21-Activated Kinase. Science 283 (5410), 2083–2085. 10.1126/science.283.5410.2083 10092231

[B26] Shi Y. Zhang L. Pu H. Mao L. Hu X. Jiang X. (2016). Rapid Endothelial Cytoskeletal Reorganization Enables Early Blood-Brain Barrier Disruption and Long-Term Ischaemic Reperfusion Brain Injury. Nat. Commun. 7, 10523. 10.1038/ncomms10523 26813496 PMC4737895

[B27] Song J. Li J. Zheng S. R. Jin Y. Huang Y. (2013). Anti-inflammatory and Immunoregulatory Effects of Yupingfeng Powder on Chronic Bronchitis Rats. Chin. J. Integr. Med. 19 (5), 353–359. 10.1007/s11655-013-1442-6 23504545

[B28] Song T. Hou X. Yu X. Wang Z. Wang R. Li Y. (2016). Adjuvant Treatment with Yupingfeng Formula for Recurrent Respiratory Tract Infections in Children: A Meta-Analysis of Randomized Controlled Trials. Phytother. Res. 30 (7), 1095–1103. 10.1002/ptr.5628 27145435

[B29] Sun H. Ni X. Zeng D. Zou F. Yang M. Peng Z. (2017). Bidirectional Immunomodulating Activity of Fermented Polysaccharides from Yupingfeng. Res. Vet. Sci. 110, 22–28. 10.1016/j.rvsc.2016.10.015 28159232

[B30] Szoták-Ajtay K. Szõke D. Kovács G. Andréka J. Brenner G. B. Giricz Z. (2020). Reduced Prenatal Pulmonary Lymphatic Function Is Observed in Clp1 (K/K) Embryos with Impaired Motor Functions Including Fetal Breathing Movements in Preparation of the Developing Lung for Inflation at Birth. Front. Bioeng. Biotechnol. 8, 136. 10.3389/fbioe.2020.00136 32211389 PMC7067749

[B31] Thiery J. P. Acloque H. Huang R. Y. Nieto M. A. (2009). Epithelial-mesenchymal Transitions in Development and Disease. Cell 139 (5), 871–890. 10.1016/j.cell.2009.11.007 19945376

[B32] Twomey E. Li Y. Lei J. Sodja C. Ribecco-Lutkiewicz M. Smith B. (2010). Regulation of MYPT1 Stability by the E3 Ubiquitin Ligase SIAH2. Exp. Cell Res. 316 (1), 68–77. 10.1016/j.yexcr.2009.09.001 19744480

[B33] Walsh N. P. Gleeson M. Shephard R. J. Gleeson M. Woods J. A. Bishop N. C. (2011). Position Statement. Part One: Immune Function and Exercise. Exerc Immunol. Rev. 17, 6–63. 21446352

[B34] Walton K. Dorne J. L. Renwick A. G. (2001). Uncertainty Factors for Chemical Risk Assessment: Interspecies Differences in Glucuronidation. Food Chem. Toxicol. 39 (12), 1175–1190. 10.1016/s0278-6915(01)00088-6 11696391

[B35] Wang J. Frost J. A. Cobb M. H. Ross E. M. (1999). Reciprocal Signaling between Heterotrimeric G Proteins and the P21-Stimulated Protein Kinase. J. Biol. Chem. 274 (44), 31641–31647. 10.1074/jbc.274.44.31641 10531372

[B36] Wang L. Wu W. Zhu X. Ng W. Gong C. Yao C. (2019). The Ancient Chinese Decoction Yu-Ping-Feng Suppresses Orthotopic Lewis Lung Cancer Tumor Growth through Increasing M1 Macrophage Polarization and CD4(+) T Cell Cytotoxicity. Front. Pharmacol. 10, 1333. 10.3389/fphar.2019.01333 31780946 PMC6857089

[B37] Wang R. Wang J. Shu J. Gu X. Li H. Zi Y. (2020). Efficacy and Safety of Yu-Ping-Feng Powder for Asthma in Children: a Protocol of Systematic Review and Meta-Analysis of Randomized Controlled Trials. Med. Baltim. 99 (1), e18551. 10.1097/MD.0000000000018551 PMC6946460 31895795

[B38] West J. B. Mathieu-Costello O. (1999). Structure, Strength, Failure, and Remodeling of the Pulmonary Blood-Gas Barrier. Annu. Rev. Physiol. 61, 543–572. 10.1146/annurev.physiol.61.1.543 10099701

[B39] Wettschureck N. Strilic B. Offermanns S. (2019). Passing the Vascular Barrier: Endothelial Signaling Processes Controlling Extravasation. Physiol. Rev. 99 (3), 1467–1525. 10.1152/physrev.00037.2018 31140373

[B40] Wojciak-Stothard B. Tsang L. Y. Paleolog E. Hall S. M. Haworth S. G. (2006). Rac1 and RhoA as Regulators of Endothelial Phenotype and Barrier Function in Hypoxia-Induced Neonatal Pulmonary Hypertension. Am. J. Physiol. Lung Cell Mol. Physiol. 290 (6), L1173–L1182. 10.1152/ajplung.00309.2005 16428270

[B41] Xu N. Dong M. Yang Y. Wang Y. Chang Y. Wan J. (2019). Integrative Transcriptomics, Proteomics, and Metabolomics Data Analysis Exploring the Injury Mechanism of Ricin on Human Lung Epithelial Cells. Toxicol Vitro 60, 160–172. 10.1016/j.tiv.2019.05.012 31103672

[B42] Yao F. Yuan Q. Song X. Zhou L. Liang G. Jiang G. (2020). Yupingfeng Granule Improves Th2-Biased Immune State in Microenvironment of Hepatocellular Carcinoma through TSLP-DC-OX40L Pathway. Evid. Based Complement. Altern. Med. 2020, 1263053. 10.1155/2020/1263053 PMC7171663 32351590

[B43] Zavorsky G. S. (2007). Evidence of Pulmonary Oedema Triggered by Exercise in Healthy Humans and Detected with Various Imaging Techniques. Acta Physiol. (Oxf) 189 (4), 305–317. 10.1111/j.1748-1716.2006.01660.x 17367400

[B44] Zhang Y. Z. Xu F. Dong J. Liang J. Hashi Y. Shang M. Y. (2012). Profiling and Identification of the Metabolites of Calycosin in Rat Hepatic 9000xg Supernatant Incubation System and the Metabolites of Calycosin-7-O-Beta-D-Glucoside in Rat Urine by HPLC-DAD-ESI-IT-TOF-MS(n) Technique. J. Pharm. Biomed. Anal. 70, 425–439. 10.1016/j.jpba.2012.06.006 22766358

[B45] Zhang J. Liu J. Ren L. Wei J. Duan J. Zhang L. (2018). PM2.5 Induces Male Reproductive Toxicity via Mitochondrial Dysfunction, DNA Damage and RIPK1 Mediated Apoptotic Signaling Pathway. Sci. Total Environ. 634, 1435–1444. 10.1016/j.scitotenv.2018.03.383 29710643

[B46] Zhong L. Gao X. Chen Y. Yu Z. Jin S. Zhu C. (2021). Roles of VE-Cadherin in Hypoxia Induced Injury of Pulmonary Microvascular Endothelial Barrier. Heart Surg. Forum 24 (4), E764–E768. 10.1532/hsf.3405 34473045

[B47] Zhou Q. Wang D. Liu Y. Yang X. Lucas R. Fischer B. (2017). Solnatide Demonstrates Profound Therapeutic Activity in a Rat Model of Pulmonary Edema Induced by Acute Hypobaric Hypoxia and Exercise. Chest 151 (3), 658–667. 10.1016/j.chest.2016.10.030 27815150

[B48] Zhou W. Wu H. Wang Q. Zhou X. Zhang Y. Wu W. (2020). Simultaneous Determination of Formononetin, Biochanin A and Their Active Metabolites in Human Breast Milk, Saliva and Urine Using Salting-Out Assisted Liquid-Liquid Extraction and Ultra High Performance Liquid Chromatography-Electrospray Ionization Tandem Mass Spectrum. J. Chromatogr. B Anal. Technol. Biomed. Life Sci. 1145, 122108. 10.1016/j.jchromb.2020.122108 32305709

