# Supplementary material for: Yu-Ping-Feng Formula Ameliorates Alveolar-Capillary Barrier Injury Induced by Exhausted-Exercise via Regulation of Cytoskeleton
Source: Front Pharmacol. 2022 Jun 24;13:891802. doi: 10.3389/fphar.2022.891802 (PMC9263595; doi:10.3389/fphar.2022.891802)
Supplement: Supplementary file 1 [file DataSheet1.pdf]

Supplementary Material

1 Supplementary Methods

1.1 HPLC/Q-TOF-MS analysis of YPF serum

Astragaloside, calycosin, formononetin, atractylenolide I, atractylenolide II, prim-O-glucosylcimifugin, and cimifugin standards were purchased from Yunnan Fengshanjian Biotechnology Co., Ltd. (Lincang, China, Series number: 2021050804, 2021032501, 2020121601, 2020090905, 2020090906, 2020051507, 2021082602). The purity of those reference standards was all more than 98%. MS-grade acetonitrile and methanol were purchased from Beijing Tongguang Fine Chemicals Company (Beijing, China). Ultrapure water was prepared by a Milli-Q system (Millipore Corporation, Billerica, MA, USA) and was used throughout.

The primary standard stock solution of Astragaloside IV (3.5 mg/ml), calycosin (3.5 mg/ml), formononetin (3.5 mg/ml), atractylenolide I (3.5 mg/ml), atractylenolide II (3.5 mg/ml), prim-O-glucosylcimifugin (3.5 mg/ml), and cimifugin (3.5 mg/ml) were prepared by dissolving in methanol. These stock solutions were further appropriately diluted to working standard solution (500 ng/ml) by ultrapure water. And 2.18 g YPF granules were dissolved by 1 ml deionized water. The standard solution and YPF solution were centrifuged at 15,000 rpm for 30 min at 4℃, the supernatant was collected, and stored at -20℃ before use.

YPF containing serum were prepared according to mentioned method. Then, 200 μl acetonitrile for protein precipitation were added to 50 μl serum sample. The mixture was then vortex-extracted for 1 min, and centrifuged for 30 min at 15,000 rpm (4℃). The supernatant was transferred to a new tube and centrifuged again. The supernatant was collected and stored at 4℃ before use.

HPLC/Q-TOF-MS analysis was carried out in State Key Laboratory of Natural and Biomimetic Drugs Large Instrument Technology Platform, Peking University Health Science Center (Beijing, China). The detection and identification were carried out on Thermo Vanquish F and Thermo Q Exactive HF-X (Thermo Fisher Scientific, Rockford, Waltham, USA). The mass range was set at 100-1000.

2 Supplementary Figures and Tables

2.1 Supplementary Figures

Supplementary Figure S1

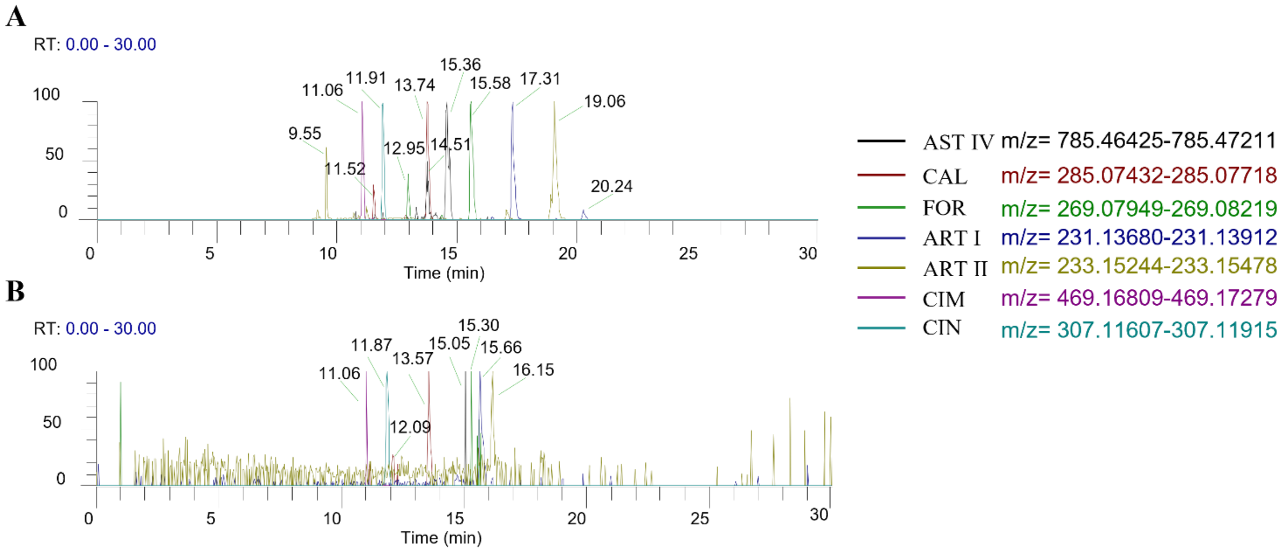

Supplementary Figure S1. Representative chromatograms of seven standards in YPF solution (A) and YPF serum (B) in positive ion mode. AST IV: Astragaloside IV; CAL: calycosin; FOR, formononetin; ART I: atractylenolide I; ART II: atractylenolide II; CIM: prim-O-glucosylcimifugin; CIN: cimifugin.

Supplementary Figure S2

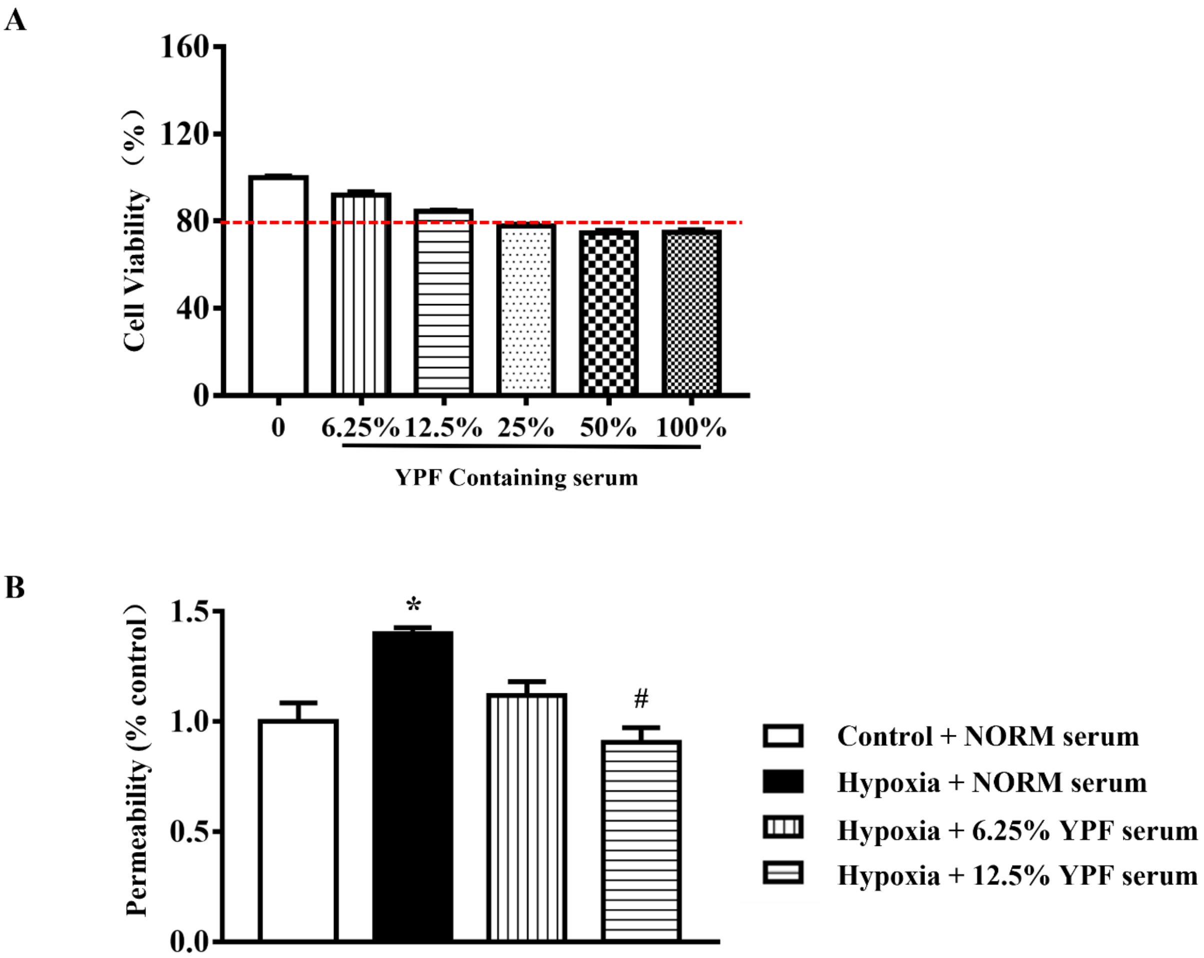

Supplementary Figure S2. Effective of YPF serum treatment on pulmonary microvascular cells viability and permeability. **(A):** The cell viability at different concentration of YPF containing serum. **(B):** Effect of YPF serum on hyperpermeability of PMVECs monolayer induced by hypoxia; n=3. Results are presented as means ± SEM. *****P< 0.05 vs. Control + NORM serum; #P< 0.05 vs. hypoxia + NORM serum.

Supplementary Figure S3

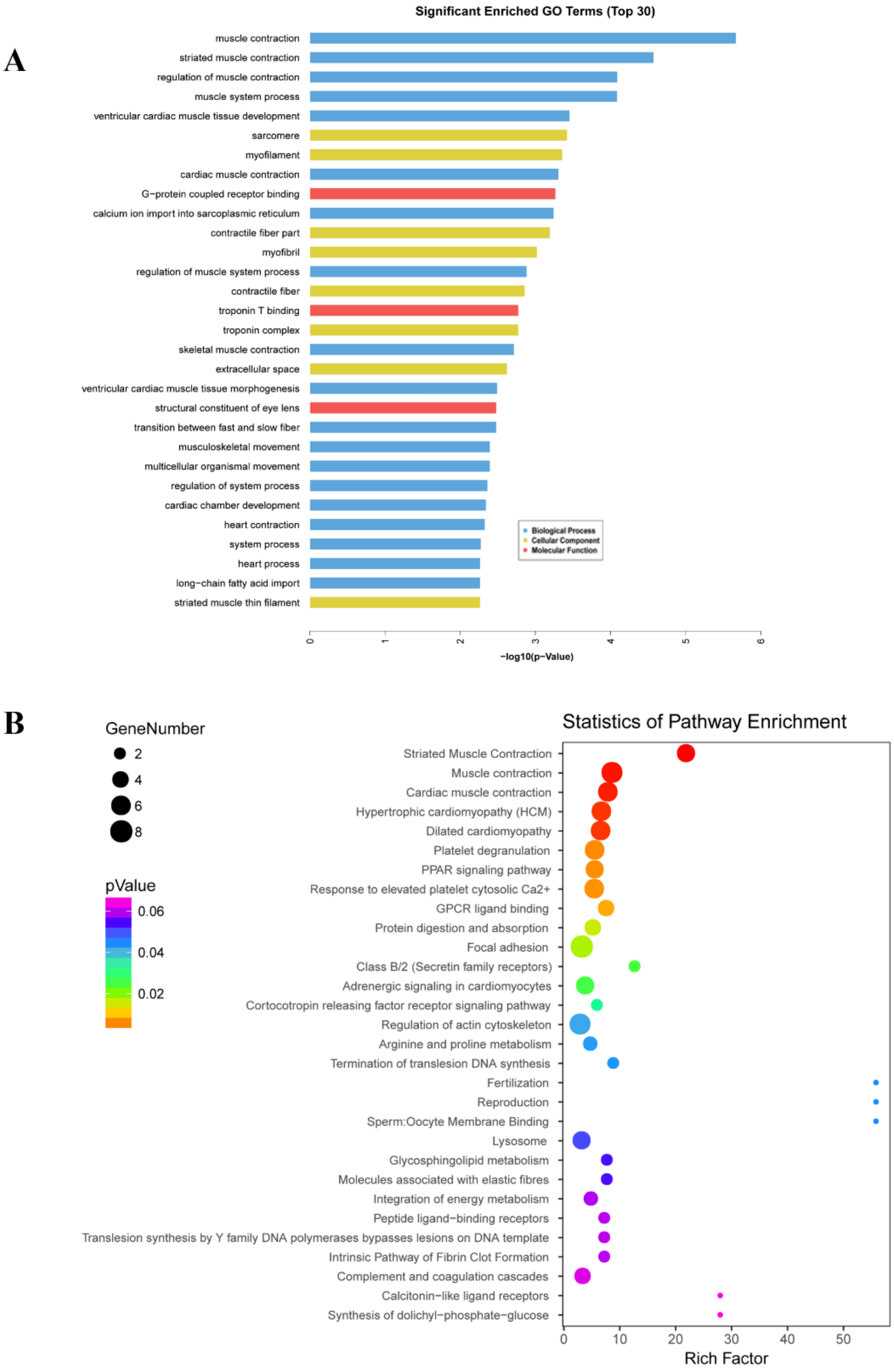

Supplementary Figure S3. Differential expressed proteins significant enriched GO Terms and pathways of exhausted-exercise group and sham group. **(A):** The significant enriched GO Terms (top30). The blue bands represent biological process; the yellow band represent cellular component; the red bands represent molecular function. **(B):** The significant pathways (top30). The color bar represents P-value. The smaller the P- value, the more reliable the enrichment of this pathway. The size of circles represents Gene numbers.

Supplementary Figure S4

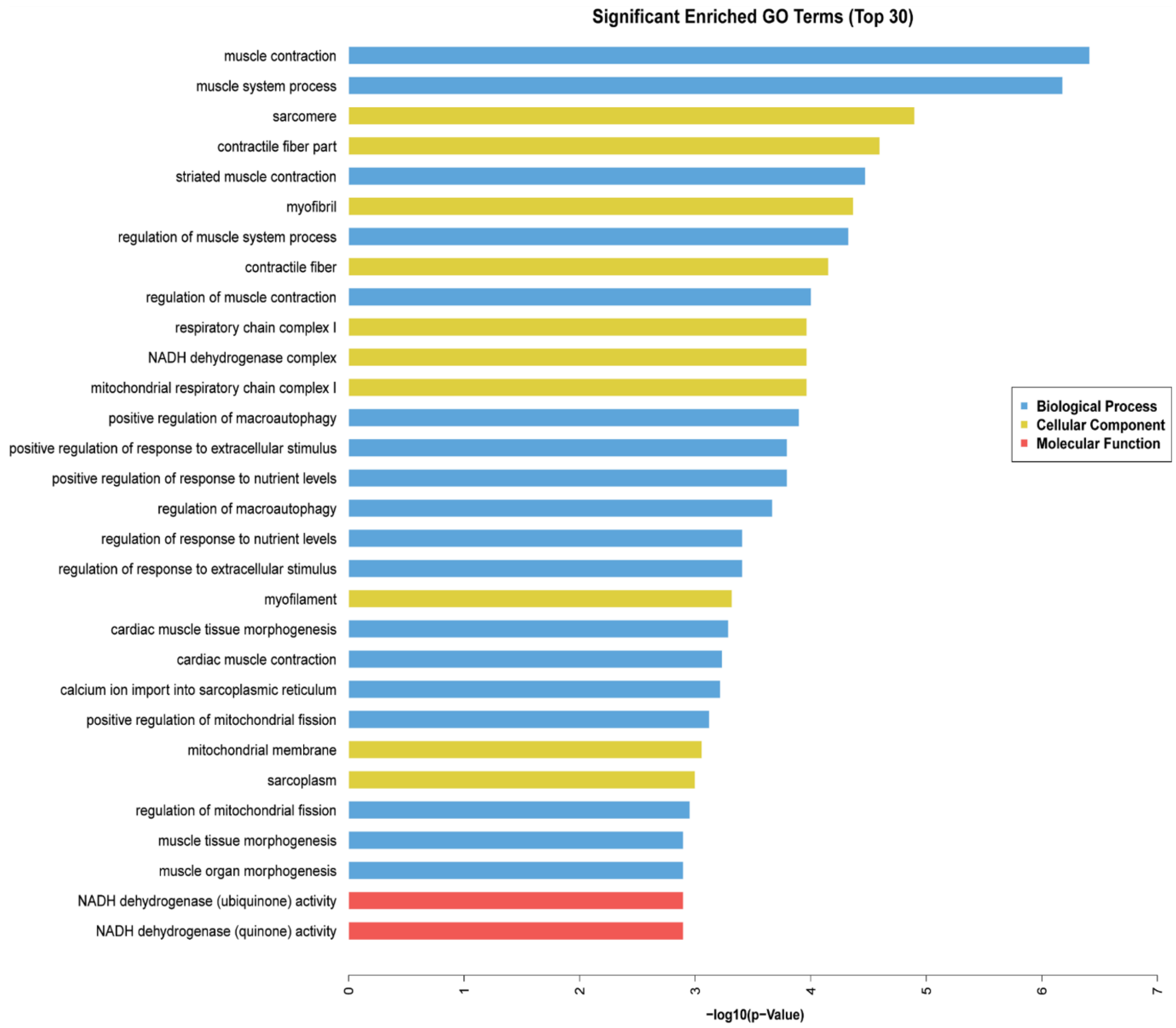

Supplementary Figure S4. Differential expressed proteins significant enriched GO Terms of exhausted-exercise + YPF group and exhausted-exercise group. The blue bands represent biological process; the yellow band represent cellular component; the red bands represent molecular function.

Supplementary Figure S5

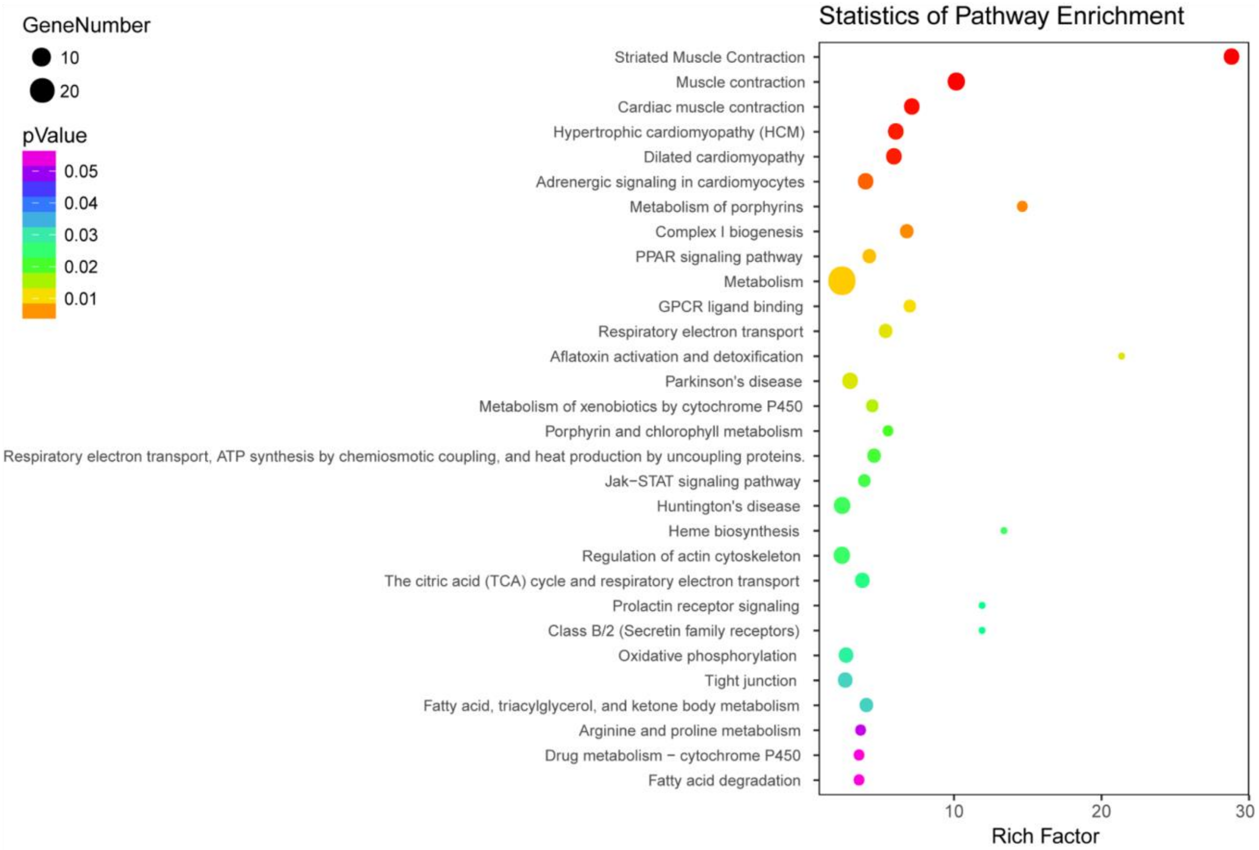

Supplementary Figure S5. Differential expressed proteins significant enriched pathways of exhausted-exercise + YPF group and exhausted-exercise group. The color bar represents P-value. The smaller the P-value, the more reliable the enrichment of this pathway. The size of circles represents Gene numbers.

Supplementary Figure S6

A

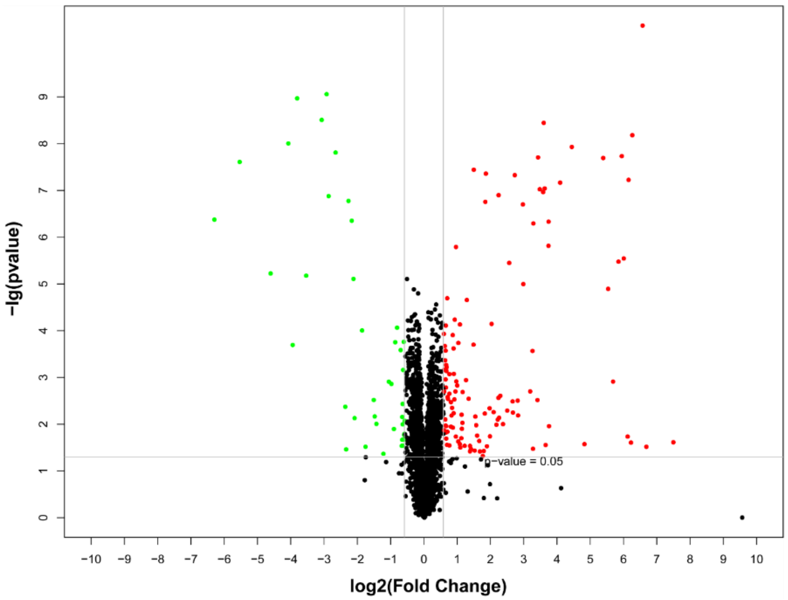

B

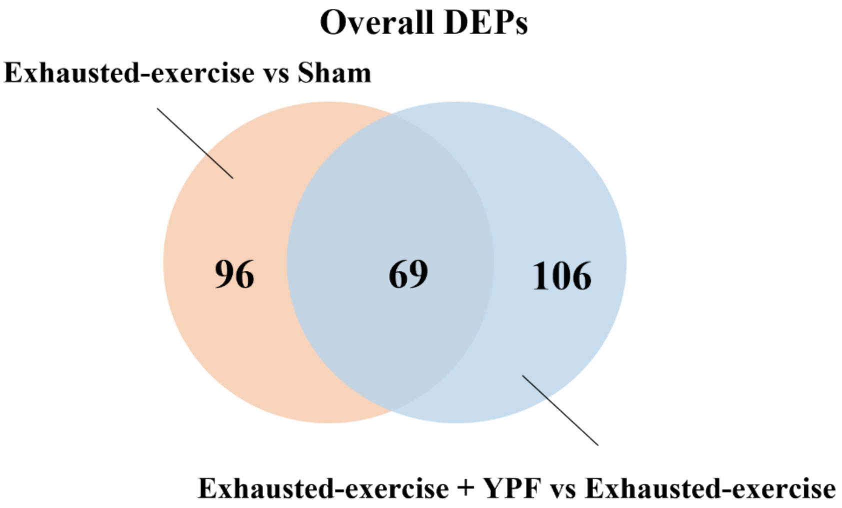

Supplementary Figure S6. (A): The volcano plot of Exhausted-exercise group and sham group. Red dots represent upregulated DEPs; green dots represent downregulated DEPs; black dots represent unchanged proteins. **(B):** Ven diagram of significantly changed proteins in exhausted-exercise vs. sham group and Exhausted-exercise group + YPF vs. Exhausted-exercise group.

Supplementary Figure S7

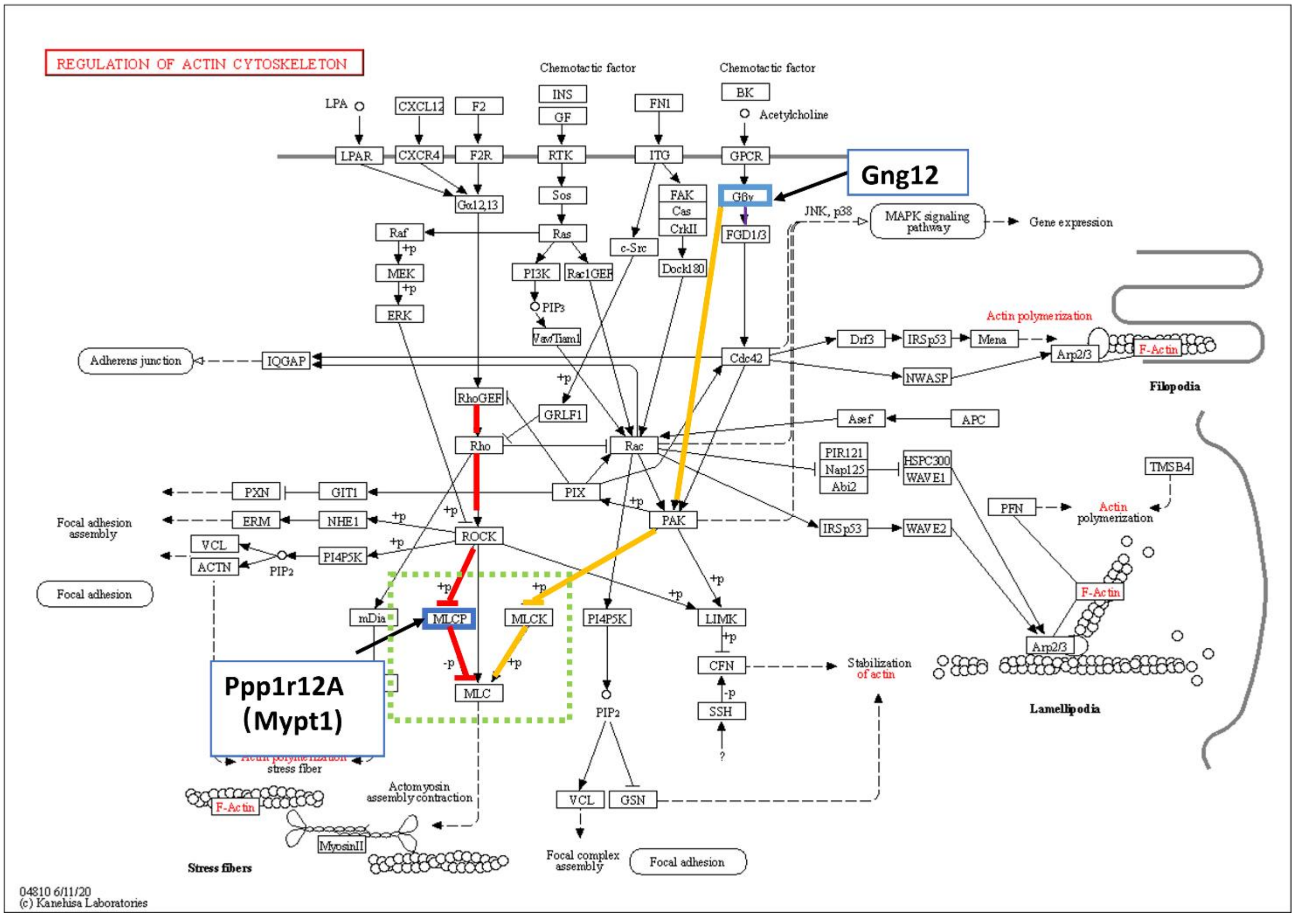

Supplementary Figure S7. Differential expressed proteins enrichment pathways that regulate actin cytoskeleton and the proteins in the pathways

Supplementary Figure S8

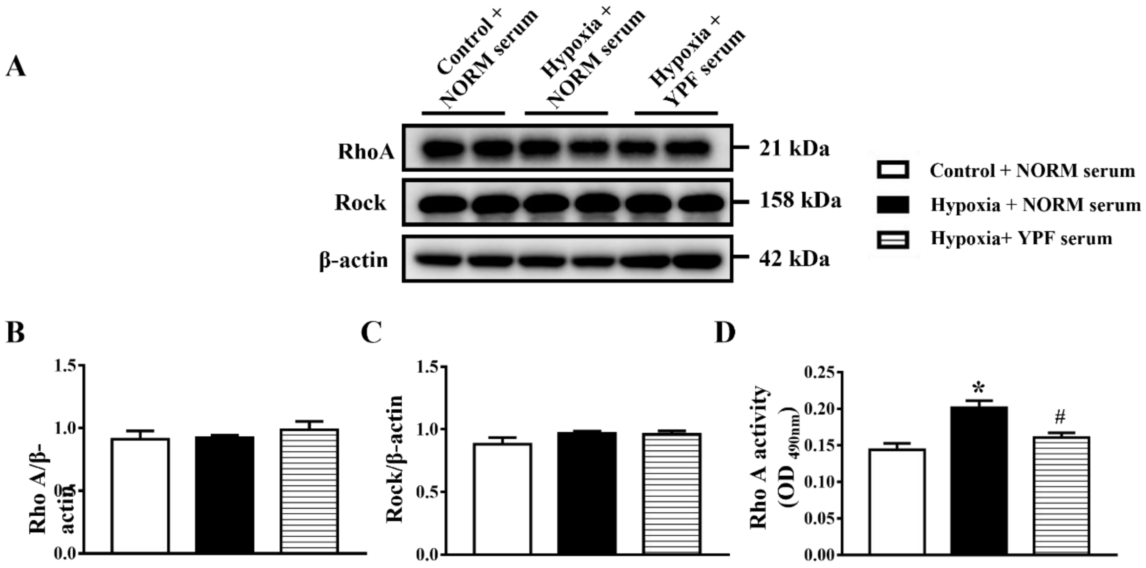

Supplementary Figure S8. (A): The representative Western bands of RhoA and Rock in lung tissue in different groups. **(B-C):** The semiquantitative analysis of RhoA **(B)** and Rock **(C)**. **(D):** The activity of RhoA in different groups. Results are presented as means ± SEM (n=4 in B and C, and n=6 in D). *****P< 0.05 vs. Control + NORM serum; #P< 0.05 vs. Hypoxia + NORM serum.

2.2 Supplementary Tables

Supplementary Table S1. The chemical constituents of YPF serum

Formula	Ion Model	RT [min]	Molecular Weight	Identification	Average Peak area
$C_{22}H_{43}NO$	[M+H]+	26.817	337.3341	Erucamide	2.19121E+10
$C_{20}H_{32}O_2$	[M+H]+	23.532	304.2401	Arachidonic acid	1.82058E+07
C_9HN	[M+H]+	13.832	129.058	Isoquinoline	1.80403E+08
$C_{10}H_{22}O_6$	[M+H]+	9.391	238.1416	PEG n5	1.03279E+08
$C_{13}H_{14}N_2S$	[M+H]+	14.242	230.0861	3-phenyl-5,6,7,8-tetrahydro [1,3] diazepino [2,1-b] [1,3] thiazole hydrobromide	1.81790E+07
$C_{17}H_{18}N_2O_3$	[M+H]+	26.243	298.1316	6-methyl-1-(2-methylphenyl)-3-propylfuro[3,2-d] pyrimidine-2,4(1H,3H)-dione	5.67384E+07
$C_{15}H_{24}O_2$	[M+H]+	20.694	218.167	NP-004713	1.01178E+08
$C_{17}H_{34}O_2$	[M+H]+	16.233	287.2823	Methyl palmitate	2.69769E+08
$C_{11}H_9NO_2$	[M+H]+	13.478	187.0633	3-Amino-2-naphthoic acid	5.43918E+07
$C_{11}H_{11}NO_2$	[M+H]+	13.834	189.079	Methyl indole-3-acetate	3.63623E+08
$C_{21}H_{30}O_4$	[M+H]+	15.182	346.2143	Corticosterone	1.19880E+08
$C_{14}H_{31}NO_2$	[M+H]+	14.179	245.2355	[Similar to: 3-[(2-pyridylthio) methyl]-4,5-dihydro-1H-pyrazol-5-one; ΔMass: 38.1888 Da]	1.05114E+08
$C_{10}H_{12}O_2$	[M+H]+	15.701	164.0838	4-Phenylbutyric acid	4.89107E+07
$C_{14}H_{15}N$	[M+H]+	11.43	197.1205	Dibenzylamine	1.45742E+07
$C_{15}H_{14}O_3$	[M+H]+	12.349	242.0943	(R)-Equol	2.83892E+07
$C_{20}H_{28}$	[M+H]+	23.589	268.219	235BBF3K97	1.35881E+08
$C_{18}H_{32}O$	[M+H]+	23.66	264.2452	2-[(5Z)-5-tetradecenyl] cyclobutanone	2.27274E+09
$C_9H_9NO_3$	[M+H]+	9.968	179.0583	Hippuric acid	8.10323E+07
$C_{21}H_{32}O_5$	[M+H]+	14.532	346.2144	Tetrahydrocortisone	2.22601E+07
$C_{20}H_{34}O_2$	[M+H]+	19.799	312.2663	Linolenic acid ethyl ester	3.02422E+07
$C_{24}H_{36}O_2$	[M+H]+	16.978	356.2713	Docosahexaenoic acid ethyl ester	4.80512E+07
$C_{24}H_{36}O_2$	[M+H]+	18.862	356.2715	Docosahexaenoic acid ethyl ester	3.74829E+07
C_9H_9NO	[M+H]+	12.295	147.0685	5-Methoxyindole	1.51015E+07

$C_{23}H_{45}NO_4$	[M+H]+	18.744	399.3349	Palmitoylcarnitine	6.23567E+07
$C_{16}H_{22}O_4$	[M+H]+	15.031	260.1412	NP-020535	2.65138E+07
$C_5H_{11}NO_2$	[M+H]+	0.949	117.0792	Betaine	5.20646E+09
$C_{33}H_{36}N_4O_6$	[M+H]+	26.242	584.2634	Bilirubin	5.43388E+07
$C_{28}H_{58}O_{15}$	[M+H]+	11.855	651.404	PEG n14	6.24438E+07
$C_{20}H_{38}O_2$	[M+H]+	25.244	292.2764	(2R)-1-(5-hydroxy-3-methylpentyl)-2,5,5,8a-tetramethyl-decahydronaphthalen-2-ol	6.97006E+08
$C_{22}H_{38}O_2$	[M+H]+	21.719	340.2977	Dihomo-γ-linolenic acid ethyl ester	2.91702E+07
$C_{18}H_{39}NO_3$	[M+H]+	15.693	317.2929	2-Amino-1,3,4-octadecanetriol	2.25783E+08
$C_{22}H_{38}N_4O_3$	[M+H]+	17.62	406.2943	[Similar to: 2-[3-chloro-2-hydroxy-4-methoxy-6-(methoxycarbonyl) phenoxy]-6-hydroxy-4-methylbenzoic acid; ΔMass: 24.2488 Da]	2.30046E+07
$C_{24}H_{49}NO_3$	[M+H]+	18.431	399.3713	[Similar to: Palmitoylcarnitine; ΔMass: 0.0364 Da]	1.03091E+07
$C_{12}H_{17}N_5$	[M+H]+	1.193	231.1471	4-(dimethylamino) benzaldehyde N-(4,5-dihydro-1H-imidazol-2-yl) hydrazone	8.10649E+07
$C_{18}H_{39}NO_4$	[M+H]+	14.433	333.2878		7.18315E+06
$C_{21}H_{40}O_3$	[M+H]+	21.333	340.2977	Glycidyl Stearate	2.01668E+07
	[M+H]+	19.796	591.2582		1.90827E+07
$C_{15}H_{33}NO$	[M+H]+	16.045	243.2562		2.10136E+07
$C_{14}H_{33}N_9O_2S$	[M+H]+	19.146	391.247		2.26426E+07
$C_4H_{15}N_7O_8S$	[M+H]+	22.876	321.0702		2.08592E+07
$C_{27}H_{44}O_3$	[M+H]+	20.861	416.329	Calcitriol	2.03553E+07
$C_{20}H_{48}ClN_7O_5S_3$	[M+H]+	19.979	597.2577		2.82652E+07
$C_{16}H_{35}NO_3$	[M+H]+	14.317	289.2615		2.66184E+07
$C_{22}H_{47}NO_3$	[M+H]+	18.094	373.3555		2.65208E+07
$C_{22}H_{48}N_3O_{12}PS_2$	[M+H]+	19.759	641.2396		3.24762E+07
$C_{20}H_{39}NO_7$	[M+H]+	21.059	405.2726		2.49706E+07
$C_{24}H_{50}NO_8P$	[M+H]+	16.044	511.3277		1.76514E+07
C24 H46 N O7 P	[M+H]+	18.009	491.3015		2.39518E+07
$C_{22}H_{46}NO_7P$	[M+H]+	20.721	467.3014	L-alpha-lysophosphatidylcholine	1.97599E+07
$C_{28}H_{59}ClNOPS_4$	[M+H]+	19.343	619.2893		2.63586E+07

$C_3H_2Cl_3NO_4P_2S$	[M+H]+	1.23	314.8245		8.85322E+06
$C_{22}H_{47}NO_4$	[M+H]+	16.98	389.3504		1.61258E+07
$C_{15}H_{33}NO$	[M+H]+	16.299	243.2562		1.64969E+07
$C_{27}H_{56}ClN_3O_5S_3$	[M+H]+	19.978	633.3053		1.33902E+08
$C_{24}H_{50}NO_8P$	[M+H]+	18.675	511.3276		1.21127E+08
$C_{35}H_{72}N_6O_{14}$	[M+H]+	12.293	800.5093		3.11295E+07
$C_{77}H_{150}N_9O_{13}P_3S_5$	[M+H]+	19.552	1661.917		9.72781E+07
$C_7H_7ClO_7S$	[M+H]+	0.939	269.9592		1.32488E+08
$C_{81}H_{162}ClN_3O_{14}P_2S_4$	[M+H]+	22.987	1626.011		5.61866E+07
$C_{19}H_{41}NO_2$	[M+H]+	17.445	315.3137		1.30115E+08
$C_{23}H_{46}NO_7P$	[M+H]+	20.253	479.3014	1-Oleoyl-2-hydroxy-sn-glycero-3-PE	9.75385E+07
$C_{21}H_{41}NO$	[M+H]+	26.007	323.3187	1-(14-methylhexadecanoyl) pyrrolidine	5.87430E+07
$C_{20}H_{30}ClN_3O_2$	[M+H]+	24.809	379.2021		4.32862E+07
$C_{27}H_{42}O_4$	[M+H]+	20.034	430.3084	Hecogenin	6.52332E+07
$C_{22}H_{21}N_5O_5$	[M+H]+	12.347	435.153		9.56974E+07
$C_{31}H_{61}N_5O_{12}$	[M+H]+	12.009	695.4302		5.69863E+07
$C_{33}H_{68}N_6O_{13}$	[M+H]+	12.157	756.4831		3.75623E+07
$C_{26}H_{39}NO_4$	[M+H]+	15.406	429.2879	Adaprolol	6.98836E+07
$C_{27}H_{44}O_2$	[M+H]+	26.123	400.3341	MFCD00010474	1.58556E+08
$C_{83}H_{142}ClN_7O_{15}P_2S_2$	[M+H]+	19.579	1637.917		1.29530E+08
$C_{23}H_{40}N_4O_3$	[M+H]+	17.619	420.3099		1.50046E+08
$C_{31}H_{59}ClN_2OS_4$	[M+H]+	22.991	638.3204		1.31874E+08
$C_{20}H_{25}ClN_2O_2$	[M+H]+	23.531	360.1598		3.69584E+07
$C_{16}H_{32}NOP$	[M+H]+	23.594	285.2218		3.28315E+07
$C_{28}H_{47}N_5O_4P_2S_2$	[M+H]+	20.871	643.2553		7.75587E+07
$C_{25}H_{47}NO_4$	[M+H]+	19.034	425.3504	MFCD22416941	3.67215E+07
$C_{16}H_{20}O_4$	[M+H]+	15.704	276.1361	2-(1-Hydroxy-2,4,6-trimethyl-3-oxo-2,3-dihydro-1H-inden-5-yl) ethyl acetate	8.16308E+07
$C_{10}H_{18}ClNO_2P_2S$	[M+H]+	20.376	313.0223		8.87443E+07
$C_6H_2ClN_2P_3S_2$	[M+H]+	1.313	293.8555		8.32364E+07
$C_{73}H_{146}ClN_9O_{17}P_2S_3$	[M+H]+	19.593	1613.917		9.52551E+07
$C_7H_3NO_4S$	[M+H]+	1.637	196.9776		7.60239E+07
$C_3H_8P_2S_2$	[M+H]+	1.244	169.9538		8.80554E+07

$C_{29}H_{60}N_6O_{11}$	[M+H]+	11.849	668.4304		2.27291E+07
C13$H_{24}C_{l2}N_6$	[M+H]+	22.764	334.1442		3.20780E+07
$C_{24}H_{50}NO_6P$	[M+H]+	21.028	479.3377	1-(1Z-hexadecenyl)-sn-glycero-3-phosphocholine	9.51001E+07
$C_{28}H_{40}N_5O_3P$	[M+H]+	19.17	525.2857		7.08368E+07
$C_{27}H_{37}N_5O_2$	[M+H]+	14.555	463.2935		2.11167E+07
$C_{26}H_{43}NO_6$	[M+H]+	15.404	465.3092	Glycocholic acid	2.46292E+07
$C_{23}H_{44}NO_7P$	[M+H]+	19.174	477.2856	1-linoleoyl-sn-glycero-3-phosphoethanolamine	4.54439E+08
$C_{25}H_{43}N_3O_6$	[M+H]+	21.72	481.3169		5.54107E+08
$C_{31}H_{65}ClNOPS_4$	[M+H]+	22.996	661.3362		5.07004E+08
	[M+H]+	1.101	148.9332		4.19975E+08
$C_{20}H_{43}NO_2$	[M+H]+	18.035	329.3292		3.50494E+08
$C_{31}H_{48}N_5O_3P$	[M+H]+	20.013	569.3482		3.27849E+08
$C_{23}H_{51}ClN_8O_3S_3$	[M+H]+	20.864	618.2941		3.94699E+08
$C_{29}H_{50}N_5O_3P$	[M+H]+	21.514	547.3639		4.18752E+08
$C_{50}H_{97}ClN_{10}O_2P_2S_4$	[M+H]+	19.602	1094.584		3.83483E+08
$C_{23}H_{53}ClN_8O_3S_3$	[M+H]+	22.993	620.3096		3.48611E+08
$C_{14}H_{31}NO$	[M+H]+	16.332	229.2406		4.94756E+07
$C_{22}H_{46}NO_7P$	[M+H]+	18.41	467.3013	L-alpha-lysophosphatidylcholine	3.91134E+08
$C_{49}H_{106}ClN_4O_7P_3S_4$	[M+H]+	19.578	1118.585		3.64431E+08
$C_{53}H_{103}ClN_4O_{11}S_3$	[M+H]+	22.994	1102.647		3.44890E+08
$C_{26}H_{54}NO_7P$	[M+H]+	22.37	523.3634	Platelet-activating factor	2.92369E+09
$C_{30}H_{50}NO_7P$	[M+H]+	19.483	567.3323	1-(4Z,7Z,10Z,13Z,16Z,19Z-docosahexaenoyl)-sn-glycero-3-phosphocholine	2.39617E+09
$C_{22}H_{38}O$	[M+H]+	25.561	318.292		1.38358E+09
$C_{28}H_{50}NO_7P$	[M+H]+	19.298	543.3322	1-arachidonoyl-sn-glycero-3-phosphocholine	2.34272E+09
$C_{22}H_{47}NO_2$	[M+H]+	19.22	357.3605		9.35329E+08
$C_{26}H_{54}NO_7P$	[M+H]+	23.004	523.3635	Platelet-activating factor	4.33735E+10
$C_{28}H_{50}NO_7P$	[M+H]+	19.55	543.3322	1-arachidonoyl-sn-glycero-3-phosphocholine	1.91944E+10
$C_{26}H_{52}NO_7P$	[M+H]+	20.858	521.348	4033747	9.39703E+09
$C_{26}H_{50}NO_7P$	[M+H]+	19.282	519.3322	1-Linoleoyl-2-Hydroxy-sn-glycero-3-PC	4.76259E+09

$C_{24}H_{48}NO_7P$	[M+H]+	18.966	493.3168	1-[(9Z)-hexadecenoyl]-sn-glycero-3-phosphocholine	7.84261E+08
$C_{23}H_{48}NO_7P$	[M+H]+	19.335	481.3168	1-Stearoyl-2-hydroxy-sn-glycero-3-PE	9.60933E+08
$C_{23}H_{52}ClN_9O_3S_3$	[M+H]+	20.376	633.3053		1.08492E+09
$C_{21}H_{48}ClN_7O_3S_3$	[M+H]+	20.863	577.2676		8.06687E+08
$C_{49}H_{95}ClN_4O_{11}S_3$	[M+H]+	20.358	1046.585		8.96989E+08
$C_{29}H_{50}O_3$	[M+H]+	26.795	446.3759	2-(3-Hydroxy-3,7,11,15-tetramethylhexadecyl)-3,5,6-trimethyl-1,4-benzoquinone	8.42542E+08
$C_{25}H_{52}NO_7P$	[M+H]+	21.524	509.3483	1-heptadecanoyl-sn-glycero-3-phosphocholine	1.50004E+09
$C_{12}H_{27}NO_2$	[M+H]+	12.388	217.2043		2.04786E+08
$C_{51}H_{106}ClN_4O_7P_3S_4$	[M+H]+	19.53	1142.585		1.78120E+08
$C_5H_{14}NO_4P$	[M+H]+	19.28	183.0661		1.85067E+08
$C_{30}H_{54}ClOPS_3$	[M+H]+	19.973	592.2785		2.34881E+08
$C_{18}H_{39}NO_2$	[M+H]+	16.846	301.298	sphinganine	2.43227E+08
$C_{35}H_{69}N_5O_{14}$	[M+H]+	12.28	783.4826		1.49320E+07
$C_{24}H_{50}ClN_3O_7S_3$	[M+H]+	19.472	623.2518		1.37466E+08
$C_2H_3C_{l3}O$	[M+H]+	1.081	147.9253	2,2,2-Trichloroethanol	1.68378E+08
$C_{45}H_{95}ClN_{10}O_{14}S_2$	[M+H]+	20.855	1098.616		1.14176E+08
$C_{30}H_{54}NO_7P$	[M+H]+	21.061	571.3639	LysoPC(22:4(7Z,10Z,13Z,16Z))	1.26061E+08
$C_{25}H_{50}NO_7P$	[M+H]+	19.885	507.3327	(2R)-1-{[(2-Aminoethoxy) (hydroxy) phosphoryl] oxy}-3-hydroxy-2-propanyl (11Z)-11-icosenoate	1.23790E+08
$C_{31}H_{64}N_6O_{12}$	[M+H]+	12.011	712.4567		3.50713E+07
C21 H38 N4 O7	[M+H]+	11.086	458.2727		7.90970E+07
$C_{28}H_{48}NO_7P$	[M+H]+	18.659	541.3167	LysoPC(20:5(5Z,8Z,11Z,14Z,17Z))	2.44714E+08
$C_{33}H_{65}N_5O_{13}$	[M+H]+	12.151	739.4566		1.87027E+07
$C_{26}H_{47}N_3O_5$	[M+H]+	21.258	481.3533		2.92516E+08
$C_5H_{14}NO_4P$	[M+H]+	19.965	183.0661		2.29875E+08
$C_{26}H_{40}N_5O_3P$	[M+H]+	19.205	501.2855		2.33220E+08
$C_{18}H_{25}ClN_2O_2$	[M+H]+	23.734	336.1598		1.39889E+08
$C_{22}H_{43}N_3OP_2S$	[M+H]+	18.015	459.262		3.83902E+07
$C_{25}H_{45}NO_4$	[M+H]+	18.302	423.3348	Linoleyl carnitine	2.29386E+07
$C_{21}H_{52}ClN_{10}PS_3$	[M+H]+	21.521	606.2941		4.01845E+07

$C_{22}H_{48}ClN_3O_7S_3$	[M+H]+	18.66	597.2364		2.96961E+07
$C_{13}H_{25}NO_2$	[M+H]+	22.612	227.1885	Cyprodenate	2.32268E+07
$C_{22}H_{45}NO_2$	[M+H]+	18.389	355.3449		4.45516E+07
$C_{20}H_{43}Cl_2N_5OP_2S$	[M+H]+	19.17	533.2051		2.47872E+07
C_{30} $H_{48}NO_4PS_5$	[M+H]+	20.875	677.1931		2.50199E+07
$C_{20}H_{39}N_5O_5S$	[M+H]+	11.427	461.2656		1.03760E+07
$C_{28}H_{54}NO_7P$	[M+H]+	21.116	547.3639	1-[(11Z,14Z)]-icosadienoyl-sn-glycero-3-phosphocholine	4.25466E+07
$C_{27}H_{42}$	[M+H]+	26.051	366.3286		2.56884E+07
$C_2H_5Cl_2NO_2P_2$	[M+H]+	1.119	206.9179		3.81849E+07
$C_{24}H_{55}ClN_5OPS_4$	[M+H]+	20.861	623.2734		2.09226E+07
$C_{37}H_{73}N_5O_{15}$	[M+H]+	12.419	827.5089		1.10958E+07
$C_4H_8N_8$	[M+H]+	0.841	168.0874		3.14036E+07
$C_{20}H_{37}NO$	[M+H]+	24.046	307.2874		1.66039E+07
$C_3H_3Cl_3P_2S_3$	[M+H]+	1.107	301.7927		2.64710E+07
$C_{26}H_{54}ClN_3O_5S_3$	[M+H]+	21.715	619.2894		2.78502E+07
$C_{19}H_{37}NO$	[M+H]+	26.816	295.2874		2.46813E+07
$C_{16}H_{14}$ N_2O_3	[M+H]+	26.242	282.1003	Bendazac	3.59835E+07
$CH_3ClO_4P_2$	[M+H]+	1.091	175.9202		3.56173E+07
$CHBrClN_2O_5PS$	[M+H]+	1.228	297.8216		2.84405E+07
$C_{27}H_{50}N_5O_4P$	[M+H]+	20.593	539.3587		4.11792E+07
$C_{31}H_{37}N_2O_7P$	[M+H]+	26.243	580.2322		4.61512E+07
$C_{22}H_{52}ClN_7O_3S_4$	[M+H]+	20.024	625.2676		2.47421E+07
$C_3H_6BrN_2O_2PS_3$	[M+H]+	1.261	307.8504		5.14901E+07
$C_{49}H_{95}ClN_4O_{11}S_3$	[M+H]+	19.961	1046.585		6.13593E+07
	[M+H]+	0.973	149.9283		6.36607E+07
$C_{41}H_{78}N_6O_8P_2$	[M+H]+	12.421	844.5354		2.60744E+07
$C_{22}H_{41}NO$	[M+H]+	26.818	335.3186	(2E,4Z)-N-Isobutyl-2,4-octadecadienamide	4.49900E+07
$C_{24}H_{44}N_5O_3P$	[M+H]+	21.341	481.3169		3.96379E+07
$C_{21}H_{39}N_5$	[M+H]+	15.774	361.3191		6.07492E+07
$C_{22}H_{46}NO_7P$	[M+H]+	18.085	467.3013	L-alpha-lysophosphatidylcholine	4.06354E+07
$C_{20}H_{31}ClN_4O_2$	[M+H]+	19.973	394.213		4.91297E+07
C25 H49 N O4	[M+H]+	19.932	427.3662	7309670	4.47643E+07
$C_{19}H_{44}ClN_7O_3S_3$	[M+H]+	18.96	549.2365		3.82783E+07

$C_{41}H_{85}N_4O_{12}PS$	[M+H]+	12.541	888.5619	1.79672E+07
$CHClN_4P_2$	[M+H]+	1.093	165.9359	3.82039E+07
$C_{14}H_{31}NO_3$	[M+H]+	12.629	261.2304	3.49469E+07
$C_{20}H_{43}NO_3$	[M+H]+	16.915	345.3242	6.15817E+07
$C_{28}H_{36}N_2O_2$	[M+H]+	25.327	432.2777	4.36512E+07
